# Comprehensive Characterization of the Integrin Family Across 32 Cancer Types

**DOI:** 10.1093/gpbjnl/qzae035

**Published:** 2024-05-09

**Authors:** Cheng Zou, Jinwei Zhu, Jiangling Xiong, Yu Tian, Yousong Peng, Edwin Cheung, Dingxiao Zhang

**Affiliations:** Hunan Key Laboratory of Animal Models and Molecular Medicine, School of Biomedical Sciences, Hunan University, Changsha 410082, China; Hunan Key Laboratory of Animal Models and Molecular Medicine, School of Biomedical Sciences, Hunan University, Changsha 410082, China; Hunan Key Laboratory of Animal Models and Molecular Medicine, School of Biomedical Sciences, Hunan University, Changsha 410082, China; Hunan Key Laboratory of Animal Models and Molecular Medicine, School of Biomedical Sciences, Hunan University, Changsha 410082, China; College of Biology, Hunan University, Changsha 410082, China; Faculty of Health Sciences, University of Macau, Macau Special Administrative Region 999078, China; Hunan Key Laboratory of Animal Models and Molecular Medicine, School of Biomedical Sciences, Hunan University, Changsha 410082, China

**Keywords:** Integrin, Pan-cancer, Tumor microenvironment, Immunotherapy, Transcriptional regulation

## Abstract

Integrin genes are widely involved in tumorigenesis. Yet, a comprehensive characterization of integrin family members and their interactome at the pan-cancer level is lacking. Here, we systematically analyzed integrin family in approximately 10,000 tumors across 32 cancer types. Globally, integrins represent a frequently altered and misexpressed pathway, with alteration and dysregulation overall being protumorigenic. Expression dysregulation, better than mutational landscape, of integrin family successfully identifies a subgroup of aggressive tumors with a high level of proliferation and stemness. The results reveal that several molecular mechanisms collectively regulate integrin expression in a context-dependent manner. For potential clinical usage, we constructed a weighted scoring system, integrinScore, to measure integrin signaling patterns in individual tumors. Remarkably, integrinScore was consistently correlated with predefined molecular subtypes in multiple cancers, with integrinScore-high tumors being more aggressive. Importantly, integrinScore was cancer-dependent and closely associated with proliferation, stemness, tumor microenvironment, metastasis, and immune signatures. IntegrinScore also predicted patients’ response to immunotherapy. By mining drug databases, we unraveled an array of compounds that may modulate integrin signaling. Finally, we built a user-friendly database, Pan-cancer Integrin Explorer (PIExplorer; http://computationalbiology.cn/PIExplorer), to facilitate researchers to explore integrin-related knowledge. Collectively, we provide a comprehensive characterization of integrins across cancers and offer gene-specific and cancer-specific rationales for developing integrin-targeted therapy.

## Graphical abstract

**Graphical abstract:**
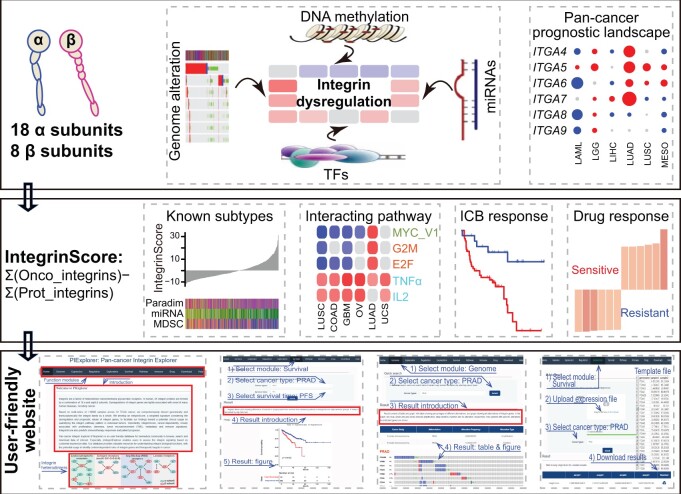

## Introduction

**Figure 1:**
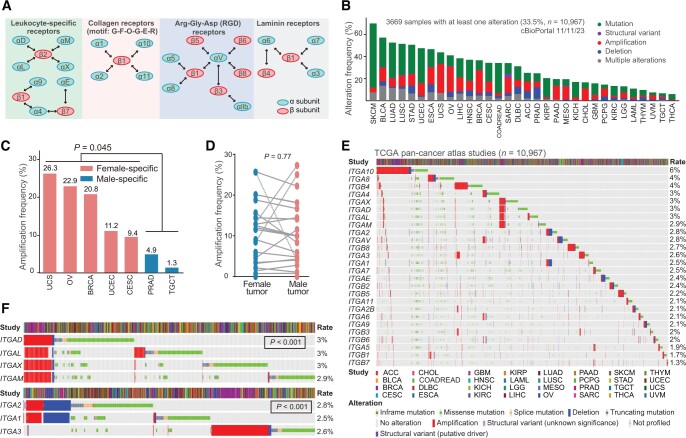
Integrin family represents a frequently altered pathway at pan-cancer level **A**. All 24 distinct integrin heterodimers formed by 26 integrin genes are classified into four groups according to cognate ligand. Bidirectional arrows are intended to demonstrate which integrins can form heterodimers. **B**. Bar plot showing the cumulative alteration frequencies of 26 integrin genes in 32 human cancers. **C**. Female-specific tumors have higher amplification frequencies than male-specific tumors. Student’s *t*-test is used for comparison. **D**. Comparison of amplification frequencies of 25 non-sex-biased cancers between females and males. Student’s *t*-test is used for comparison. Female-specific (BRCA, CESC, OV, UCEC, and UCS) and male-specific (PRAD and TGCT) tumors are not included. **E**. Heatmap illustrating distinct alteration frequencies of 26 integrin genes in 32 human cancers. **F**. Co-occurrence and mutually exclusion pattern of seven integrin genes. Data shown on the right are alteration frequencies. *P* values were calculated by the Fisher’s exact test. TCGA, The Cancer Genome Atlas; ACC, adrenocortical carcinoma; BLCA, bladder urothelial carcinoma; BRCA, breast invasive carcinoma; CESC, cervical squamous cell carcinoma and endocervical adenocarcinoma; CHOL, cholangiocarcinoma; COAD, colon adenocarcinoma; DLBC, diffuse large B-cell lymphoma; ESCA, esophageal carcinoma; GBM, glioblastoma multiforme; HNSC, head and neck squamous cell carcinoma; KICH, chromophobe renal cell carcinoma; KIRC, clear cell renal clear cell carcinoma; KIRP, papillary renal cell carcinoma; LAML, acute myeloid leukaemia; LGG, lower-grade glioma; LIHC, hepatocellular carcinoma; LUAD, lung adenocarcinoma; LUSC, lung squamous cell carcinoma; MESO, mesothelioma; OV, ovarian serous adenocarcinoma; PAAD, pancreatic adenocarcinoma; PCPG, phaeochromocytoma and paraganglioma; PRAD, prostate adenocarcinoma; READ, rectal adenocarcinoma; SARC, adult soft tissue sarcoma; SKCM, skin cutaneous melanoma; STAD, stomach adenocarcinoma; TGCT, testicular germ cell tumor; THCA, thyroid carcinoma; THYM, thymoma; UCEC, uterine corpus endometrial carcinoma; UCS, uterine carcinosarcoma; UVM, uveal melanoma.

Integrins are a family of heterodimeric transmembrane glycoprotein receptors consisting of α and β subunits. In humans, 26 integrins are formed by sophisticated combinations of 18 α and 8 β subunits (**Figure 1**A). Upon binding to adjacent ligands, integrins act as adhesion receptors and bidirectionally transmit biochemical signals across the plasma membrane, thus enabling cells to generate a rapid response to intracellular and extracellular cues. Given that integrins can adhere to nearly all extracellular matrix (ECM) components, they can remodel the extracellular environment. Based on the types of ligands, integrins are divided into four categories (Figure 1A) [1,2]. Especially, different integrins can bind to the same ligand, and the same integrin can recognize and bind to multiple distinct ligands, creating an intricate signaling network of integrin signalosome. Biologically, the integrin signalosome represents a dynamic cascade responsible for various cellular “outside-in” and “inside-out” signal transmission and thus regulates a spectrum of important biological processes such as cell growth, survival, and migration [3]. Notably, dysregulation of the integrin signalosome is tightly associated with the onset of many human diseases, including cancer [3].

Integrins are involved in almost every step of cancer development and progression (including metastasis and drug resistance) [1,4–6]. For example, comparative genomic analysis of tumors and matched normal tissues indicated that *ITGA7* mutations causing protein truncations are associated with tumor initiation in prostate, brain, and liver tissues [7]. Studies on hematological and solid cancers have shown that integrins play a key role in regulating proliferation, migration, and invasiveness by interacting with multiple oncogenic pathways [5,6,8]. In addition, integrins have emerged as valuable therapeutic targets for drug development. Recently, we have provided our perspective on how the integrin signalosome modulates cancer stemness, and summarized current clinical trials evaluating integrin-targeted drugs in solid tumors [4]. At present, numerous integrin-targeted drugs are under investigation in more than 130 clinical trials [1,4].

Immunotherapy has revolutionized cancer treatment as evidenced by the discovery and successful clinical application of immune checkpoint blockades (ICBs) targeting CTLA4, PD-1, and PD-L1 [9]. However, a majority of patients do not benefit from ICB owing to a number of mechanisms that block the cytotoxicity of T cells, such as the immune escape of cancer cells, inactivation of antitumor immune cells, and tumor microenvironment (TME) remodeling. Because integrins possess the intrinsic property of modulating cell–cell and cell–ECM interactions, they are thought to be tightly associated with the abovementioned immunosuppressive mechanisms [10–12], providing a rationale for combining integrin inhibitors with ICB to achieve a better antitumor efficacy. In support, integrin αvβ6 can deactivate T cells and drive immune evasion and thus inhibition of αvβ6 in combination with ICB has been shown to improve immunotherapy responses in colorectal [13] and breast cancers [14].

Tumor tissue is extremely heterogeneous, with genes and/or pathways exerting functions in a cancer-dependent manner. Therefore, understanding integrin-mediated roles and interactomes in distinct tumor types is necessary for developing more effective antitumor strategies. Despite recent advances of characterizing individual integrins’ role in cancer, a comprehensive and tumor type-specific investigation of all 26 integrins is still lacking. Using The Cancer Genome Atlas (TCGA) database, which has profiled the genomic, transcriptomic, and epigenomic landscapes of nearly 10,000 primary tumors across 32 cancer types (Table S1) [15], we systematically analyzed and compared the genomic and transcriptomic profiles of integrin pathway as a whole, as well as integrin interactomes, across distinct cancers. The results showed that dysregulation of integrins was attributed to the synergistic effects of genomic alteration, DNA methylation-mediated and microRNA (miRNA)-mediated epigenetic regulation, and transcription factor (TF)-mediated transcription, and played generally an oncogenic role in most of the cancers. The expression profile of 26 integrin genes consistently identified a subgroup of aggressive tumors featured by a high level of proliferation and stemness. An integrinScore, a weighted signature based on the dysregulation and prognostic values of integrin genes, was developed to facilitate our findings toward a potential clinical usage by quantifying the integrin pathway pattern in individual tumors. Besides a close correlation of integrinScore to patients’ outcomes and previously reported molecular subtypes in many cancers, integrinScore can be used to predict responses to ICBs. By mining the Connectivity Map (CMap) and the Genomics of Drug Sensitivity in Cancer (GDSC) drug sensitivity databases, we discovered a number of potential drugs that may interfere with the suppression or activation of integrins. Finally, we built a user-friendly database Pan-cancer Integrin Explorer (PIExplorer) (http://computationalbiology.cn/PIExplorer), a valuable resource for the biomedical community to browse, search, and download data of interest.

## Results

### Genomic alteration landscape of integrin genes in human cancers

cBioPortal was used to analyze genomic aberrations in all 26 genes encoding human integrins (hereinafter referred to as integrin genes) across 32 cancer types (Figure 1A) [16]. Aggregately, the integrin family as a group of cell adhesion molecules represented a frequently altered pathway in 32 cancers. On average, 33.5% of the tumors contained at least one alteration in one integrin gene, with skin cutaneous melanoma (SKCM, 70.0%) and thyroid carcinoma (THCA, 6.0%) having the highest and lowest mutation frequencies, respectively (Figure 1B; Table S2). Detailed analysis of distinct alteration types revealed several interesting patterns. First, mutations occurred most often, followed by amplification, in a majority of cancers, especially in SKCM (58.1%) (Figure 1B; Table S2), consistent with a high mutation burden for SKCM among human cancers [17]. Second, amplification was observed more frequently in five female-specific cancers [uterine carcinosarcoma (UCS), ovarian serous adenocarcinoma (OV), breast invasive carcinoma (BRCA), uterine corpus endometrial carcinoma (UCEC), and cervical squamous cell carcinoma and endocervical adenocarcinoma (CESC)] than in two male-specific cancers [prostate adenocarcinoma (PRAD) and testicular germ cell tumor (TCGT)] (Figure 1C), while no significant difference was found between these two groups for mutation (*t*-test, *P* = 0.089) and deletion (*t*-test, *P* = 0.58). Moreover, the frequency of amplification was not notably different among the remaining non-sex-biased 25 cancers (Figure 1D). These results strongly supported that amplification in integrin genes may be associated with sex hormone sensitive cancer types. Third, deletion occurred predominantly in PRAD (10.5%), stomach adenocarcinoma (STAD, 7.3%), and esophageal carcinoma (ESCA, 7.1%), indicating the tumor suppressive role and loss-of-expression of integrin genes in these cancer types (also see below). Fourth, despite the overall high alteration rate of integrin genes, most individual integrin genes had a low mutation frequency, with an average alteration rate of 3% (Figure 1E). Interestingly, we found that several integrin genes were co-mutated. For example, *ITGAD*, *ITGAL*, *ITGAX*, and *ITGAM* were co-amplified owing to their same location at Chr16p11.2 (*P* < 0.001) (Figure 1F). Copy number variants (CNVs) at the Chr16p11 region has been associated with neurodevelopmental disorders and invasive BRCA [18]. Similarly, owing to distinct genomic locations, *ITGA1* and *ITGA2* both resided at Chr5q11.2 were co-altered (*P* < 0.001) but mutually exclusive to *ITGA3*, which is located at Chr17q21.33 (Figure 1F). Integrin proteins function through heterodimers and are generally grouped into four categories (Figure 1A) based on their ligand types [1,4]. *ITGAD*, *ITGAL*, *ITGAX*, and *ITGAM* all dimerize with *ITGAB2* to form leukocyte-specific receptors. Therefore, the co-amplification of these four α integrins indicates a functional similarity and/or redundancy and highlights their important roles in modulating tumor immunity. Although all coupled with *ITGB1*, *ITGA3* functions as a laminin receptor, whereas *ITGA1* and *ITGA2* function as collagen receptors. A mutually exclusive mutation pattern between *ITGA3* and *ITGA1/2* suggested their different roles in cancer development. Fifth, a detailed comparison of the mutational landscape of individual integrin genes across 32 cancers revealed that they altered in both a tumor-specific and gene-specific manner (Figure S1A). For example, in terms of the mutation rate, *ITGA10* (6%) and *ITGB7* (1.3%) are the highest and lowest altered ones, respectively (Figure 1E). In terms of alteration types, *ITGA10* had high amplification rates in hepatocellular carcinoma (LIHC, 9.7%), BRCA (9.2%), and bladder urothelial carcinoma (BLCA, 9.0%), whereas it predominantly mutated in SKCM (7.8%). In terms of cancer types, *ITGA10* and *ITGA5* represented the most frequently amplified genes in cholangiocarcinoma (CHOL), whereas other integrin genes were rarely amplified (Figure S1A). Moreover, integrin genes are frequently, but rarely, depleted in PRAD and CHOL, respectively. In total, these results indicated that integrin genes are frequently mutated in cancer. Importantly, the genomic alteration landscape of the integrin family was overtly heterogeneous at multiple layers, suggesting the diversity in the mechanisms through which integrins influence cancer development.

Classification of pan-cancer patients based on the presence or absence of genomic alterations in integrin genes showed that the altered group had shorter overall survival (OS) and progression-free survival (PFS) time than the non-altered group (Figure S1B and C), indicating a general protumorigenic role of the genomically altered integrin family. Particularly, when we “zoomed in” on individual genes, we found that: (1) alteration of most individual integrin genes was not prognostic across 32 cancers (only 3.0% for OS and 3.1% for PFS) (Figure S1D and E), which may be attributed to limited alteration events in distinct cancer types; and (2) prognostic integrin genes based on genomic alterations displayed cancer type dependency, as many genes consistently played a protumorigenic role in head and neck squamous cell carcinoma (HNSC) but a tumor-suppressive role in UCEC (Figure S1D and E). Collectively, these results suggested that the prognostic power of integrin genes based solely on genomic alterations was inadequate to stratify cancer cohorts clearly.

### Expression dysregulation of integrin genes across cancers

**Figure 2:**
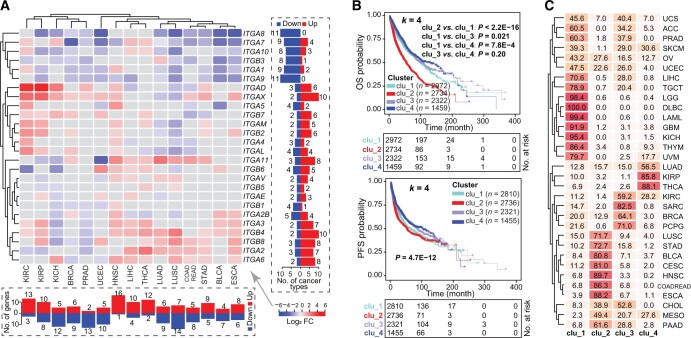
Integrin genes are dysregulated in human cancers and their general expression patterns are correlated with patients’ outcomes **A**. Heatmap showing differential expression of integrin genes between tumors and adjacent normal tissues. The right and bottom panels summarize the numbers of significantly changed integrins at gene and tumor levels, respectively. Gray rectangles in the heatmap denote non-significant results at the cutoff of absolute of Log_2_ FC > 1 and FDR < 0.05. Unsupervised clustering method is used. **B**. Four integrin expression patterns correlated with patients’ OS (the top two panels) and PFS (the bottom two panels). **C**. Sample distribution in the four clusters. Data and color shown in boxes are the percentage and intensity of samples classified in corresponding clusters, respectively. FC, fold change; FDR, false discovery rate; OS, overall survival; PFS, progression-free survival.

We next examined the global misexpression of the integrin family by interrogating the transcriptomes of 15 TCGA cancer types, which have at least 10 matched tumor and normal samples for each cancer type. The results showed that, globally, all integrin genes were dysregulated in the 15 cancers, with each gene misexpressed in at least 4–12 cancers (**Figure 2**A; Table S3). Notably, both cancer type-specific and gene-specific dysregulation were seen. Lung squamous cell carcinoma (LUSC) had the most deregulated integrin genes (21 genes), followed by papillary renal cell carcinoma (KIRP; 18 genes), chromophobe renal cell carcinoma (KICH; 17 genes) and HNSC (17 genes) (Figure 2A, bottom). Particularly, HNSC, THCA, and clear cell renal clear cell carcinoma (KIRC) represented cancers with preferentially up-regulated integrin genes, whereas LUSC, PRAD, UCEC, and KICH showed an opposite pattern (Figure 2A, bottom). At the individual gene level, *ITGA8* and *ITGA9* were down-regulated in 11 out of the 15 cancers, whereas *ITGAX*, *ITGB4*, and *ITGA6* were more frequently up-regulated in tumors relative to adjacent normal tissues (Figure 2A, right). These results together highlighted a heterogeneity in the dysregulation of integrin genes across human cancers, reflecting distinct roles played by integrins in different pathological contexts. Of note, consistent with the co-amplification profile of *ITGAD*, *ITGAL*, *ITGAX*, and *ITGAM* (Figure 1E), we observed similar dysregulation patterns for them in cancers (belonging to one sub-cluster) (Figure 2A), validating our analytic pipeline.

Cancer-specific dysregulation of integrin genes indicated potentially a population stratification ability according to their expression profile. Using unsupervised consensus clustering, we grouped all 32 pan-cancer samples according to the transcriptome of 26 integrin genes into four subclusters (*k* = 4; namely clu_1 to clu_4) (Table S4), with patients in clu_2 showing the shortest OS and PFS followed by those in clu_1 (Figure 2B). To verify that the *k*-value of 4 enabled the ideal stratification (Figure S2A), we also tested *k* = 3 and *k* = 5 and observed similar results, with clu_2 exhibiting clinically aggressive characteristics (Figure S2B–G). Importantly, regardless of the cutoff of the subcluster number (*k* = 3, 4, or 5), the dysregulation of integrin genes identified, convergently, the same subgroup of tumors with worse outcomes (Figure S2D and G). With regard to the classification of cancer types in the four subclusters, diffuse large B-cell lymphoma (DLBC; 100%), acute myeloid leukemia (LAML; 99.4%) and lower-grade glioma (LGG; 98.4%) were mainly included in clu_1, whereas HNSC (89.7%), ESCA (88.2%), and colon and rectal adenocarcinoma (COADREAD; 86.3%) were predominantly included in clu_2 (Figure 2C), in line with the cancer-dependent dysregulation of integrin genes. Furthermore, different subclusters were dominated by distinct integrin genes (Figure S2H). For example, *ITGB4*, *ITGB6*, *ITGA2*, and *ITGA6* predominantly expressed in clu_2, whereas *ITGA9*, *ITGB3*, and *ITGA3* mainly expressed in clu_4. Considering the fact that *ITGB4*, *ITGA2*, and *ITGA6* were frequently up-regulated in cancer *vs*. normal tissues (Figure 2A), our results suggest potential protumorigenic roles for them. Consistently, oncogenic roles have been reported for these three integrins in different cancer types [4].

### Mechanisms underpinning transcriptional dysregulation of integrin

**Figure 3:**
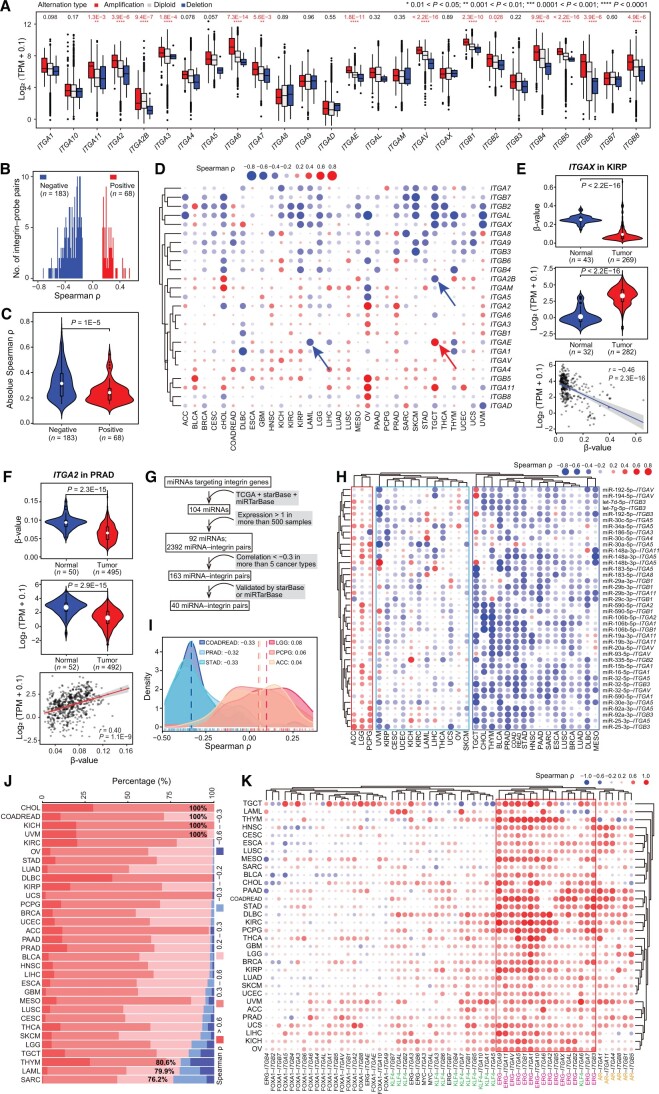
Mechanisms underlying integrin dysregulation in tumors **A**. Box plot showing integrin expression in tumor samples with amplification, diploid, and deletion alterations, with *P* values shown on the top. Within the plot, the center lines represent median values, box edges are 75th and 25th percentiles, and dots denote the outliers. **B**. Bar plot showing the number of integrin–probe pairs with varied Spearman ρ values. 183 and 68 significantly negatively and positively correlated pairs are shown, respectively. **C**. Violin plot showing the absolute Spearman ρ values in negatively and positively correlated pairs. The Wilcoxon test is used. Within the violin plot, the center white dots represent mean values, box edges are 75th and 25th percentiles, and dots denote the outliers. **D**. Spearman correlation of DNA methylation in promoters with expression of integrin genes. Blue and red arrows denote three examples of distinct correlations between integrin–probe pairs. *ITGA10* is not included due to the absence of probe in its promoter. **E**. and **F**. DNA methylation partially contributes to *ITGAX* (E) and *ITGA2* (F) dysregulation in tumors. Top and middle are the violin plots showing DNA methylation in promoter and expression of integrin gene in tumors and normal tissues, respectively; at the bottom are dot plot showing Spearman correlation between DNA methylation in promoter and expression of integrin gene. Within the violin plots, the center white dots represent mean values, box edges are 75th and 25th percentiles, and dots denote the outliers. **G**. Workflow for correlation analysis between miRNAs and integrin genes. **H**. Spearman correlation of expression of miRNAs and integrin genes. Three correlation patterns (negative-relationship, mixed-relationship, and positive-relationship) are identified by unsupervised clustering method. **I**. Density plot showing Spearman ρ values between miRNAs and integrin genes in extreme cancers. Three positive-correlation dominant cancers (LGG, PCPG, and ACC) and three negative-correlation dominant cancers (COADREAD, PRAD, and STAD) are shown. **J**. Bar plot showing cumulative percentage of significant correlated TF–integrin pairs with distinct ranges of Spearman ρ values. The cutoff of Spearman ρ ≥ 0.2 and *P* < 0.05 is considered significant. The cancers are ordered by the percentage of positively correlated pairs. Several extreme cancers are labeled with the percentage values. **K**. Spearman correlation of expression of TFs and integrin genes. The relationships between ERG and integrin genes are overwhelmingly positive and boxed by a red square. In (D), (H), and (K), positive and negative correlations are colored in red and blue, respectively. The size of dots indicates the absolute Spearman ρ. TF, transcription factor; TPM, transcripts per kilobase of exon model per million mapped reads; miRNA, microRNA; ρ, Spearman correlation coefficient.

Genomic (Figure 1) and transcriptional (Figure 2) aberrations underscore the significance of the integrin family in human cancers. Then, a question that arises is what mechanisms drive their expression dysregulation. We considered both genetic and epigenetic mechanisms. First of all, genomic alterations are major drivers of cancer initiation and progression [19]. CNVs are frequently linked to altered gene expression [20]. Indeed, amplification and deletion were generally correlated with gain and loss of messenger RNA (mRNA) expression, respectively (**Figure 3**A). Notably, the mutational burden of individual integrin genes was quite low in pan-cancer samples (Figure 1E), suggesting that genomic alterations could only partly explain the misexpression of them and thus the existence of other regulatory mechanisms.

Next, we examined the impact of DNA methylation on gene expression. By focusing on the DNA methylation probes within promoter regions of integrin genes, we observed a significant negative correlation for most integrin–probe pairs in pan-cancer [absolute Spearman correlation coefficient (ρ) ≥ 0.15] (Figure 3B), suggesting, as expected, that DNA methylation (measured as probe β-value) suppresses gene expression. Moreover, the absolute Spearman ρ values of negative integrin–probe pairs were larger than those of positive pairs (Figure 3C), indicating a severity in inhibiting, rather than enhancing, integrin gene expression by DNA methylation. A positive correlation between DNA methylation and elevated gene expression was also reported [21]. The relationship between DNA methylation and gene expression of individual integrin genes exhibited gene-dependent and cancer-dependent patterns (Figure 3D; Table S5). For example, DNA methylation levels of *ITGA2B* and *ITGAE* were negatively and positively correlated with their expression in TGCT, respectively. Also, at gene level, an opposite relationship was observed for the same *ITGAE* gene in different cancer types (*e.g.*, TGCT and LAML) (Figure S3A). Integrative analysis of the transcriptome and DNA methylome suggests that DNA methylation explains the dysregulation of some but not all integrin genes. *ITGAX* had a lower promoter methylation level in, and thus displayed overexpression in, tumor *vs*. normal tissues in KIRP (Figure 3E). For *ITGA2*, a positive correlation between its expression and methylation was observed, in line with both lower expression and methylation level in tumor *vs*. normal tissues in PRAD (Figure 3F). Similarly, inconsistency was observed between expression and methylation levels of *ITGA11* in LIHC (Figure S3B) and *ITGB7* in THCA (Figure S3C). Both elevated expression and methylation were observed for *ITGB7* in THCA, indicating that methylation may enhance expression, but instead, an inverse correlation between them was found (Figure S3C). Collectively, our data illustrate a cancer-dependent effect of DNA methylation on regulating integrin gene expression, and the inconsistency between integrin misexpression and mis-methylation highlights the involvement of other regulatory layers.

Third, we investigated the miRNA-mediated repression of integrin genes, as dysregulated miRNAs contribute to tumor development via reshaping cancer transcriptome [22–24]. To comprehensively profile the miRNA regulome for integrin genes, we first constructed a strict analysis workflow (Figure 3G) to narrow down to the final 40 miRNA–integrin pairs. A majority of the miRNA–integrin pairs in TCGA cohorts had a negative relationship, with a median Spearman ρ of −0.16 (Figure S3D; Table S6). The relationship was validated in an independent dataset (Figure S3E). Subsequently, utilizing unsupervised clustering, we obtained three pan-cancer subgroups based on the negative-relationship, mixed-relationship, and positive-relationship of miRNA–integrin pairs (Figure 3H). By a cutoff of absolute Spearman ρ ≥ 0.3, COADREAD had the highest number (*n* = 28) of negatively correlated miRNA–integrin pairs, followed by PRAD (*n* = 25) and STAD (*n* = 24), whereas LGG, adrenocortical carcinoma (ACC), and phaeochromocytoma and paraganglioma (PCPG) had a higher number of positively correlated miRNA–integrin pairs (Figure 3H and I, Figure S3F). Interestingly, PRAD and BLCA, both of which originate from the genitourinary system, exhibited similar miRNA–integrin correlation patterns (Figure 3H). However, COADREAD and LGG showed nearly opposite correlation patterns (Figure 3H), as the same set of miRNA–integrin pairs had opposite effects on gene expression (Figure S3G and H). This cancer type specificity may be attributed to the intrinsic abnormalities in miRNA biogenesis and maturation processes in distinct cancer types. For example, malfunction of the post-transcriptional maturation of miRNAs caused by the aberrant nuclear localization of DICER leads to widespread dysregulation of miRNAs. Consequently, the resulting miRNAs do not efficiently target their targets in glioblastoma [25]. In this study, a substantial proportion of targeting genes (83.3% for miR-32-5p and 64.0% for miR-148a-3p) was found to be positively rather than negatively correlated with their expression in LGG (Figure S3I). As such, this global miRNAome abnormality may contribute to the twisted miRNA–integrin relationship seen in LGG *vs*. other cancer types with negative miRNA–integrin relationship (Figure 3H and I).

Last, we investigated a TF-based mechanism, as oncogenic TFs drive nearly all cancer-related biological processes by controlling transcription [26,27]. In a human TF database, 495 human TFs have been annotated with potential targets [28]. TFs that potentially target integrin genes were screened, and the top 10 TFs with the most integrin targets were selected for further analysis (Figure S3J). By a cutoff of absolute Spearman ρ ≥ 0.2, a majority of significant TF–integrin pairs were positively correlated (Figure 3J; Table S7), suggesting an overall transcriptional promotion on integrin genes by these TFs regardless of cancer types. Many TFs are oncogenic drivers. For example, myelocytomatosis (MYC) is a notorious oncoprotein overexpressed in most of human cancers [29], and androgen receptor (AR) and forkhead box A1 (FOXA1) are pioneer factors in PRAD [30]. Focusing on five well-studied oncogenic TFs [*i.e.*, AR, FOXA1, ETS transcription factor (ERG), Kruppel like factor 4 (KLK4), and MYC], we profiled their potential regulation on integrin genes, and found a positive correlation between the expression of most TF–integrin pairs (Figure 3K; Table S7). Although FOXA1 and MYC had a less significant correlation with integrin genes, ERG showed an overwhelmingly positive correlation with them in pan-cancer, pointing ERG as an essential upstream TF of integrins. AR is expressed in multiple tissues, and it is positively correlated with six integrin genes in PRAD and also with other integrin genes in other cancer types (*e.g.*, HNSC and CESC), indicating a weak cancer type specificity. KLF4 is a renowned stemness factor [31]. The moderate positive correlation between KLF4 and integrin genes in pan-cancer implied that KLF4 may impact cellular stemness by, at least partially, regulating integrin genes. In support, evidence has implicated integrins in regulating cancer stemness [4]. We found several negatively correlated TF–integrin pairs, such as FOXA1–*ITGB4* in PRAD and FOXA1–*ITGA3/ITGA5* in HNSC (Figure 3K). Consistent with their negative correlation of expression, an opposite expression pattern was observed for these FOXA1–integrin pairs in both PRAD and HNSC (Figure S3K). These results were validated in two independent datasets, GSE157548 for PRAD and the Chinese Glioma Genome Atlas (CGGA) for LGG [32]. Consistent with the results observed in TCGA cohorts, TFs were found to play a role in modulating integrin expression in the two validation datasets (Figure S3L and M). Our data together illustrate a notion that there is an array of TFs, instead of a single TF, to cooperatively determine an integrin profile in each cancer type.

In summary, we conclude that an intricate network of mechanisms (including genomic alterations, DNA methylation, miRNA, and dominant oncogenic TFs) is responsible for the transcriptional dysregulation of integrin genes in pan-cancer and that such a network may function in a cancer-dependent or gene-dependent manner. Notably, these mechanisms represent different regulatory layers of integrin gene regulation. Conceivably, cross-talk between them is expected. For example, after integrating the associations among miRNAs, TFs, and integrin genes, we depicted a reciprocal and cross-regulatory map (Figure S3N, left). Specifically, both miR-29b-3p and MYC can regulate *ITGB1*, and MYC can in turn regulate miR-29b-3p (Figure S3N, right). To experimentally validate the impact of different mechanisms (*i.e.*, DNA methylation, miRNA, and TF) on integrin gene expression, we utilized AR^+^ LNCaP as a model representing PRAD. In our pan-cancer analysis, miR-92a-3p was identified as a hub miRNA regulating integrin family (Figure 3H). Thus, 5′-Aza (a DNA methylation inhibitor), miR-92a-3p overexpression, and enzalutamide (an AR inhibitor) were used to disrupt the global DNA methylation, miRNA expression, and a prominent TF’s activity, respectively. As for a readout, we chose the indicated 12 integrin genes whose expression was readily detectable in LNCaP cells. Results showed that expression of many of the integrin genes was altered after 5′-Aza (Figure S3O), miR-92a-3p overexpression (Figure S3P), or enzalutamide treatment (Figure S3Q), demonstrating that modulation of these regulatory mechanisms could result in dysregulation of integrin family in cancer cells.

### Integrin expression patterns classify pan-cancers into four clusters with distinct clinical features

**Figure 4:**
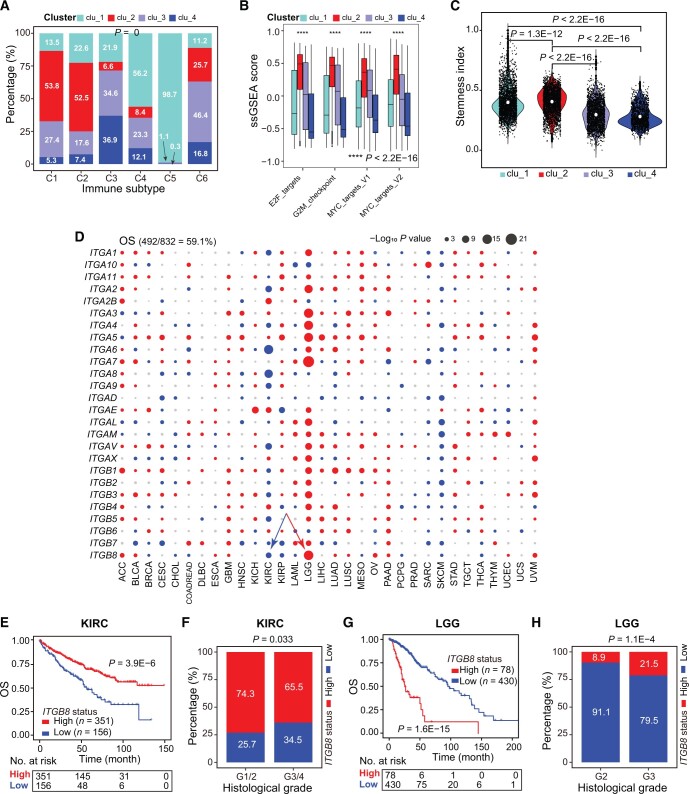
General expression patterns of integrin genes are correlated with cancer hallmarks and patient’s survival **A**. Distribution of six previously reported immune subtypes in four clusters. *P* value was calculated by the chi-square test. **B**. Box plot showing the activity of proliferation-related signatures among four clusters. Within the plot, the center lines represent median values, box edges are 75th and 25th percentiles, and dots denote the outliers. *P* values were calculated by the Wilcoxon test. **C**. Violin plot showing the stemness index among four clusters. Within the plot, the center white dots represent mean values. *P* values were calculated by the Wilcoxon test. **D**. Summary of OS time analysis results of integrin genes across cancers. Red, blue, and gray dots indicate worse, better, and non-significant results, respectively. The size of dots indicates −log_10_*P* value. Data in the parenthesis (top left) denote the rate of dysregulation associated with OS. **E**. Kaplan–Meier plot illustrating *ITGA8* as a favorable gene associated with patient OS in KIRC. **F**. Comparison of the percentage of distinct histological grades between *ITGA8* high-expression and low-expression groups in KIRC. *P* value was calculated by the chi-square test. **G**. Kaplan–Meier plot illustrating *ITGA8* as an unfavorable gene associated with patient OS in LGG. **H**. Comparison of the percentage of distinct histological grades between *ITGA8* high-expression and low-expression groups in LGG. *P* value was calculated by the chi-square test.

To molecularly characterize our four integrin subclusters (Figure 2B), we utilized two recently reported pan-cancer classification systems. Thorsson et al. have integrated major immunogenomics methods to classify > 10,000 TCGA samples into six immune subtypes (namely C1–C6); of which, C1 and C2 are characterized by enhanced proliferation [33]. Here, we found that more than half of the tumors originally grouped in C1 (53.8%) and C2 (52.5%) were classified as clu_2 in our study (**Figure 4**A). Notably, gene set variation analysis (GSVA) against proliferation-related signatures showed that tumors in clu_2 possessed the highest scores (Figure 4B), highlighting a proliferative nature. Of the four integrin subclusters, clu_2 and clu_1 had worse outcomes according to the survival analysis (Figure 2B). Stemness is a hallmark of cancer and parallels with cancer progression [34]. Recently, a pan-cancer stemness feature correlated with oncogenic dedifferentiation and metastasis was identified via machine learning based on large-scale molecular data [35]. Interestingly, we found that clu_1 and clu_2 displayed a higher stemness index among the four subclusters (Figure 4C), in line with their worse clinical outcome (Figure 2B). Collectively, the transcriptome of integrin family could stratify pan-cancer patients into four subclusters with distinct clinical and molecular features. Particularly, tumors in clu_2 exhibited higher proliferation and stemness and thus had worse outcomes.

Furthermore, we assessed the prognostic values of the 26 individual integrin genes in pan-cancer. We found that 59.1% and 58.1% of dysregulated integrin genes were frequently correlated with patients’ OS (Figure 4D) and PFS (Figure S4A) in distinct cancer types (Table S8), respectively. Globally, integrin genes with higher expression acted as risk factors across cancers (Figure 4D), especially in LGG, ACC, and uveal melanoma (UVM). In contrast, higher expression of many integrin genes was uniformly correlated with prolonged OS/PFS in KIRC, adult soft tissue sarcoma (SARC), and SKCM, indicating them as protectors in specific cancer types. Interestingly, for the same integrin genes, a heterogeneous correlation with patients’ OS/PFS was also observed. For example, *ITGB8* was found to play a tumor-suppressive role in KIRC, as patients with higher *ITGB8* expression displayed longer OS time and lower tumor stages (Figure 4E and F). However, conversely, *ITGB8* was found to play an oncogenic role in LGG (Figure 4G and H). *ITGA5* was associated with a poor prognosis in most (19/32) cancer types, whereas its higher expression was correlated with a good prognosis in ACC, indicating its protective role (Figure S4B). A similar pattern was also observed for *ITGB7* (Figure S4C). Interestingly, in cancers originating from the same tissue (such as KICH, KIRC, and KIRP), the same integrin genes were found to play opposing roles in different contexts. For example, *ITGAE* played an oncogenic role in KICH and KIRC but a tumor-suppressive role in KIRP (Figure S4D). Although a majority of integrin genes were prognostic, a gene-specific and cancer-specific pattern was observed, necessitating the development of a strategy that considers and simplifies this heterogeneity before translating the prognostic values of integrins into the clinic.

### IntegrinScore molecularly separates aggressive primary tumors from indolent ones

The widespread dysregulation of integrin genes possessed prognostic values either at the family (Figure 2B) or individual gene (Figure 4D) level across cancers. However, the directional heterogeneity in misexpression (up-regulation or down-regulation) and prognostic values (risk factor or protector) across cancers did not allow us to assess and integrate all integrin genes in individual patients using traditional methods like GSVA [36] or Z-normalization [37] for potential clinical application. Consequently, we adopted a method reported in our previous study [38] to construct integrinScore, a weighted scoring model that considers both the prognostic value (risk and protective genes as positively and negatively weighted genes, respectively) and normalized expression value (up-regulation or down-regulation in a given tumor type) of all integrin genes in a tumor sample (see Materials and methods). Survival analysis revealed that integrinScore well separated patients into two groups, with the integrinScore-high group having shorter survival than the integrinScore-low group (Figure S5A). Using the same model, we extended our analysis to other datasets spanning six cancer types and observed consistent results (Figure S5B). Thus, integrinScore can be used as a biomarker to uniformly stratify a cohort of tumors with differential aggressiveness.

**Figure 5:**
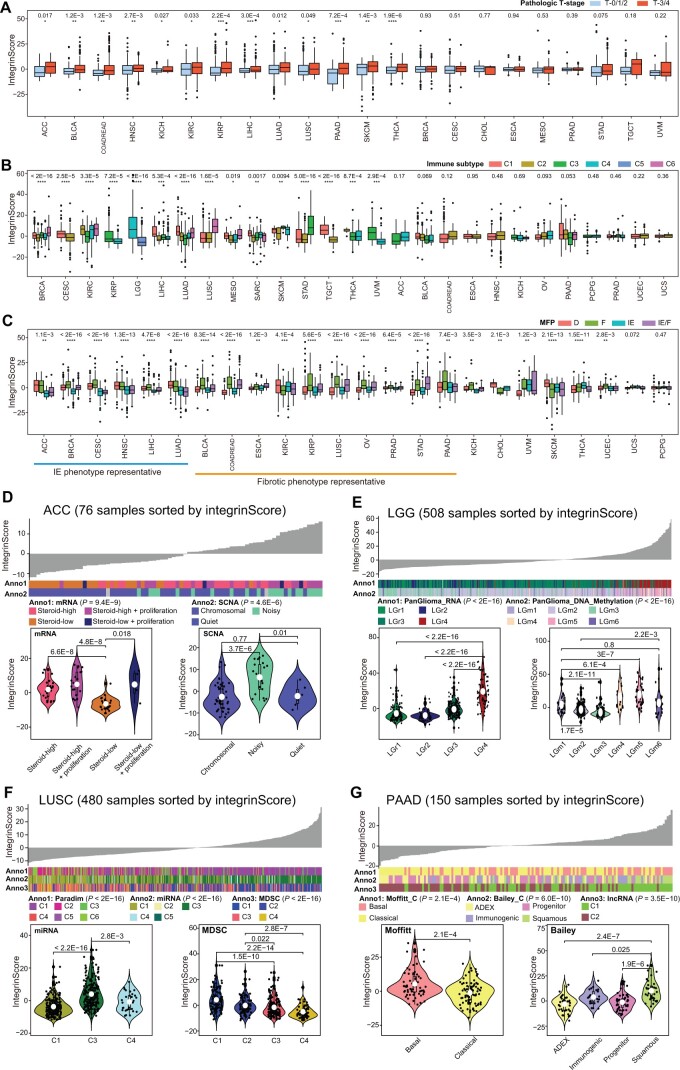
Molecular and clinical features associated with integrinScore in distinct cancers **A**. Box plots showing integrinScores in higher (T-3/4) and lower (T-0/1/2) pathological T-stages across cancers. **B**. and **C**. Box plots showing integrinScores among six immune subgroups (B) and four TME subgroups (C) across cancers. **D**.–**G**. Top are the overviews of the association between known molecular subtypes and integrinScores in ACC (D), LGG (E), LUSC (F), and PAAD (G). Columns represent samples sorted by integrinScore from low to high. At the bottom are violin plots showing integrinScores among distinct molecular subtypes. Within the box plots and violin plots, the center lines represent median values, box edges are 75th and 25th percentiles, and white dots in violin plots denote mean values. In (A–C), *P* values were calculated by the Kruskal–Wallis text (*, *P* < 0.05; **, *P* < 0.01; ***, *P* < 0.001; ****, *P* < 0.0001); In (D–G), *P* values were calculated by the Wilcoxon test. TME, tumor microenvironment; IE/F, immune-enriched and fibrotic; IE, immune-enriched but nonfibrotic; F, fibrotic; D, immune-depleted; mRNA, messenger RNA; SCNA, somatic copy number alteration; PanGlioma_RNA, Pan-glioma RNA subtype; PanGlioma_DNA_Methylation, Pan-glioma DNA methylation subtype; MDSC, myeloid-derived suppressor cell; lncRNA, long non-coding RNA; MFP, molecular functional portrait; ADEX, aberrantly differentiated exocrine.

To validate these findings, we next examined the association between integrinScore and various previously reported clinical and/or molecular cancer features. Globally, integrinScore was significantly higher in tumors with advanced pathological T-stages (**Figure 5**A), in line with a risk role of integrinScore in most of the cancers (Figure S5A). Recently, Bagaev et al. categorized TCGA tumors into four TME subtypes (based on a set of 29 knowledge-based functional gene signatures): immune-enriched and fibrotic (IE/F), immune-enriched but nonfibrotic (IE), fibrotic (F), and immune-depleted (D) subtypes [39]. Examination of Thorsson’s six immune subtypes [33] and Bagaev’s four TME subtypes [39] with respect to integrinScore, we noticed several intriguing points. First, integrinScore significantly fluctuated across the immune (15/27) (Figure 5B) and TME (22/24) (Figure 5C) subtypes in a majority of cancers, indicating a TME-reshaping ability of integrins. Second, the C1 subtype was originally reported to be more proliferative [33]; however, we observed that the C1 tumors exhibited higher integrinScore only in a limited number of cancers [*e.g.*, LIHC and lung adenocarcinoma (LUAD)], with some cancers in C1 (*e.g.*, LUSC and STAD) displaying a lower integrinScore (Figure 5B). These results were validated using two proliferation-related signatures. The correlation between integrinScore and proliferation was found to be positive in LIHC and LUAD but negative in LUSC and STAD (Figure S5C), indicating a cancer-dependent relationship between integrinScore and proliferation. Third, integrinScore was associated with a representative IE-phenotype in some cancers but with a F-phenotype in other cancers (Figure 5C). In the IE-phenotype cancers, such as ACC (Figure S5D, left), similar and lower integrinScores were observed in the IE and IE/F subgroups relative to D and F. Combined stratification of ACC based on integrinScore and IE-phenotype further identified an aggressive subgroup characterized by a high integrinScore and non-IE phenotype (Figure S5D, right). In contrast, in F-phenotype cancers, took OV as an example, tumors in F and IE/F showed comparable high integrinScore values relative to D and IE (Figure 5C, Figure S5E, left), with the combination of high integrinScore and F-phenotype indicating an aggressive tumor subgroup (Figure S5E, right). Notably, not all cancer subtypes can be separated by integrinScore, as there was a minimal difference in integrinScore among several cancers when classified based on immune or TME features (Figure 5B and C). For example, no differences were observed in integrinScore among UCS and PCPG subtypes.

To assess the clinical utility of integrinScore, we focused on individual cancer types. A strong correlation was observed between integrinScore and several known molecular subtypes in the majority of the TCGA cancers (Figure 5D–G, Figure S5F–W). In ACC tumors, steroid hormones rendered a hostile environment to immune infiltrating cells and thus promoted tumor progression [40]. Consistently, we found that tumors with lower steroid levels tended to have lower integrinScore (Figure 5D). A previous study reported that a subgroup of ACC tumors (noisy group) characterized by a significantly higher number of chromosomal breaks caused by frequent arm-level CNVs possessed worse prognosis [40]. Here, we found that tumors in the noisy group had a higher integrinScore (Figure 5D). In LGG, Ceccarelli et al. categorized more than 1100 diffuse gliomas into distinct molecular subtypes by multidimensional omics [41]. They reported that the PanGlioma_RNA cluster LGr4 and PanGlioma_DNA_methylation cluster LGm4/5/6 contained a large proportion of high-grade tumors with a poor prognosis. Consistently, higher integrinScore values were seen in LGr4 and LGm4/5/6 tumors (Figure 5E). In addition, classical (CL) and mesenchymal (ME) tumors, which have worse outcomes, had higher integrinScores than neural (NE) and pro-neural (PN) ones (Figure S5F, left). Combination of integrinScore with the CL or ME subtype further stratified and thus identified a subgroup of aggressive tumors (Figure S5F, right). Analysis of data from advanced glioblastoma multiforme (GBM) yielded similar results (Figure S5G).

Based on ∼ 1000 variable pathway features, Campbell et al. clustered > 1300 squamous cancers (including LUSC) into 6 subclusters (PARADIGM C1–C6), with C1 and C2 being enriched for active immune-related (*i.e.*, IE-phenotype) and proliferation pathways, respectively [42]. LUSC was an F-phenotype representative cancer with F and IE/F subtypes showing higher integrinScore (Figure S5H, left). Given that the C1 subtype had higher integrinScore (Figure S5H, middle), we speculated that it contained more IE/F tumors. Projecting tumors in PARADIGM subclusters into four TME subtypes revealed that C1 had the highest proportion (29.2%) of IE/F tumors (Figure S5H, right), validating our hypothesis. Furthermore, the proliferative C2 subtype had lower integrinScores, which was consistent with the negative correlation between proliferation and integrinScore in LUSC (Figure S5C and H, middle). IntegrinScore also showed a strong correlation with the miRNA cluster, with C3 having the highest values (Figure 5F) and more F and IE/F tumors (Figure S5H, right). Myeloid-derived suppressor cells (MDSCs) are a heterogeneous group of immunosuppressive cells originating from the myeloid lineage, and their abundance in the TME is associated with worse outcomes and drug resistance [43,44]. Based on 49 MDSC-related gene expressions, squamous cancers were clustered into four subclusters (MDSC C1–C4), with C1 and C2 being the MDSC-inflamed subclusters associated with poor outcomes [42]. In our study, integrinScore of tumors gradually decreased from the MDSC C1 to C4 (Figure 5F), and the same trend was observed for the proportion of F and IE/F tumors (Figure S5H, right), indicating that the F-phenotype played an oncogenic role in LUSC.

A dense fibrotic stroma is the definitive feature of pancreatic adenocarcinoma (PAAD), which serves as a barrier to capturing the intrinsic molecular features of epithelial cancer cells. In Moffitt’s study, a blind source separation method was utilized to classify PAAD tumors into basal and classical subtypes, in which basal subtype molecularly resembled basal breast tumors [45]. Consistently, basal PAAD was found to have worse outcomes and higher integrinScores (Figure 5G). Bailey et al. used mRNA expression profiles to categorize PAAD into four subtypes, with the squamous subtype exhibiting the worst outcome [46]. Here, we found that squamous tumors had the highest integrinScore (Figure 5G). Immunogenically, squamous tumors were characterized by the activation of CD4^+^ regulatory T cells and inhibition of antigen-presenting cells, leading to immunosuppression in the TME [46]. This phenotype was also reflected by the higher proportion of tumors in the D and F subtypes (Figure S5I).

In addition to abovementioned cancer types, we also extended our analysis to several other TCGA cancer types, and found that integrinScore was also tightly correlated with multiple known subtypes defined by genomic [*e.g.*, B-Raf proto-oncogene (BRAF) mutation for THCA and somatic copy-number alteration (SCNA) for TGCT], transcriptomic [*e.g.*, mRNA for KIRP and UCEC, miRNA for COADREAD and mesothelioma (MESO), and long non-coding RNA (lncRNA) for KIRC], epigenetic (*e.g.*, methylation for THCA and SKCM), and histological (*e.g.*, UCEC) features (Figure S5J–W). Collectively, our data establish integrinScore as a general marker capable of separating patients into clinically distinguishable subgroups. Importantly, the cancer type-specific and consistent relationship of integrinScore with distinct previously reported molecular subtypes highlights the functional diversity of integrins in tumor development and progression.

### Molecular pathway characterization of pan-cancer integrinScore subclusters

**Figure 6:**
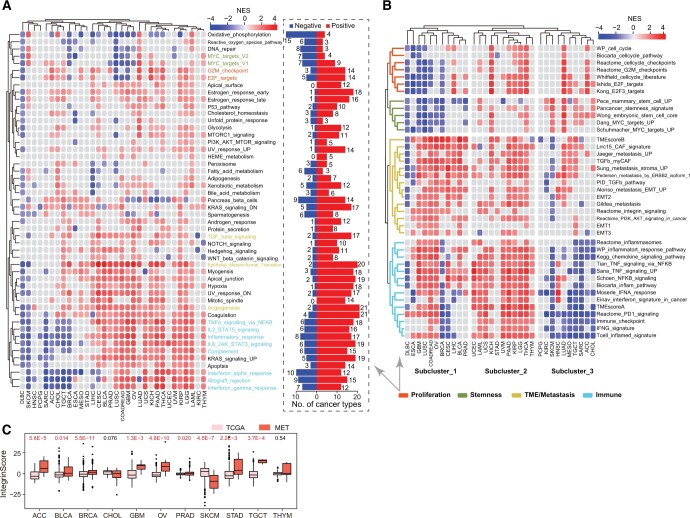
IntegrinScore is correlated with cancer hallmarks across cancers **A**. Left: heatmap showing NES of 50 cancer hallmarks across cancers. Right: bar plot showing the number of cancer types in which each signature significantly enriches. **B**. Heatmap showing NES of curated signatures across cancers. Unsupervised clustering method is used for subgroup identification. In (A) and (B), red, blue, and gray boxes denote positive, negative, and non-significant enrichment, respectively. Proliferation-, stemness-, TME/metastasis-, and immune-related signatures are labeled with different colors. **C**. Box plot showing integrinScores in primary and metastatic tumors across cancers. *P* values were calculated by the Wilcoxon test. NES, normalized enrichment score.

IntegrinScore uniformly identified a clinically aggressive cancer subcluster with distinct molecular features in a cancer type-specific manner prompting us to explore the biological pathways associated with the expression profile of integrin genes across cancers. We first identified the differentially expressed genes (DEGs) between integrinScore-high *vs*. integrinScore-low groups and observed substantial variations in DEG number among different cancer types, ranging from 463 in CHOL to 8462 in PAAD (Figure S6A). Certain cancer types had a higher ratio of up-regulated* vs.* down-regulated DEGs (*e.g.*, 25.4-fold for STAD, 13.2-fold for KIRC, and 9.4-fold for ESCA), whereas some cancer types had a higher ratio of down-regulated *vs.* up-regulated DEGs (*e.g.*, 13.0-fold for HNSC, 5.1-fold for CHOL, and 4.3-fold for UCEC). These results indicate that integrins reprogram the transcriptome in a cancer-specific manner. The abovementioned DEGs were subjected to gene set enrichment analysis (GSEA) to identify key biological processes. Globally, 50 hallmark gene sets were either positively or negatively enriched in integrinScore-high (*vs*. low) groups in a cancer-dependent manner (**Figure 6**A; Table S9). In most cancers, proliferation-related pathways (*e.g.*, E2F targets and G2M checkpoint) were positively enriched in integrinScore-high subclusters (Figure 6A). However, stemness-related signatures, such as MYC targets, displayed both positive and negative enrichment in integrinScore-high groups in a similar number of cancer types, indicating intrinsic biological differences among distinct cancer types. Notably, we noted that TME-related pathways [*e.g.*, transforming growth factor (TGF)-β signaling, epithelial mesenchymal transition (EMT), and angiogenesis], and immune signatures [*e.g.*, tumor necrosis factor (TNF)-α signaling via nuclear factor-kappa B (NF-κB), inflammatory response, and IL6–JAK–STAT3 signaling], were predominantly positively, rather than negatively, enriched in integrinScore-high groups across cancers.

To validate these findings, we performed GSEA against a curated set of gene signatures (Table S10). Overall, the enrichment pattern was similar (Figure 6B) to that shown in Figure 6A across cancers. For example, stemness signatures showed overall negative enrichment in DLBC, BRCA, GBM, and LUSC (Figure 6B, Figure S6B; Table S10), indicating intrinsic biological heterogeneity between these and other cancer types. Consistently, a negative association between RNAs (a stemness feature extracted using transcriptomic data via machine learning) and survival was also reported in GBM [35]. In this study, only a few stemness genes, such as *SOX2*, *TWIST1*, and *CD34*, were aberrantly expressed [absolute fold change (FC) ≥ 2] in more than five cancer types (Figure S6C), indicating that integrins may function downstream of key stemness factors. Unsupervised clustering grouped proliferation-related and stemness-related signatures into one subcluster, highlighting an indistinguishable relationship between them in tumor evolution (Figure 6B).

Intriguingly, TME-related (except immune cells) and metastasis-related signatures, including integrin signaling, showed similar enrichment patterns across cancers (Figure 6B), indicating that the role of integrins in TME remodeling may promote metastasis [5,6]. EMT endows cancer cells with morphological plasticity, which facilitates their migration through barriers, consequently leading to invasion and metastasis [47]. EMT signatures predominantly positively enriched in integrinScore-high tumors across cancers (Figure 6B, Figure S6B). Notably, in bulk RNA sequencing (RNA-seq) data, these EMT signatures should be reflected largely by the stromal compartment within the TME, but not necessarily by epithelial cancer cells. Indeed, we examined the correlation between integrinScore and the mRNA expression of many known EMT markers and revealed a preferential positive correlation between integrinScore and mesenchymal markers in the majority of cancer types (Figure S6D). Especially, this correlation was more significant in adenocarcinomas including STAD, COADREAD, PRAD, and PAAD, indicating intrinsic similarities in metastasis among adenocarcinomas originating from different organs. On the contrary, a negative correlation was observed between mesenchymal markers and integrinScore in DLBC, SKCM, and KIRC, which was consistent with the negative enrichment of EMT signatures in the integrinScore-high groups in these cancers (Figure 6B). The strong correlation between integrinScore and TME/metastasis signatures indicates that integrinScore could be used to predict metastasis (Figure 6B, Figure S6D). To strengthen this idea, we integrated TCGA cohorts (mainly containing localized primary tumors) with the MET500 dataset (containing transcriptomes of 500 metastatic samples from 22 organs) [48]. Pairwise comparison revealed that, in most cancer types, integrinScores were significantly higher in metastatic *vs*. primary tumor samples (Figure 6C). Notably, primary SKCM samples had higher integrinScores than metastatic samples, which may be attributed to the source of most SKCM samples being metastatic lymph nodes. Similar integrinScores were observed between primary and metastatic samples in CHOL and thymoma (THYM), which was consistent with the generally insignificant enrichment of EMT/metastasis signatures in integrinScore-high *vs*. integrinScore-low groups in these cancers (Figure 6B).

In summary, these results suggest that integrins interact with diverse biological pathways centric to proliferation, stemness, EMT, metastasis, and immunity in a context-dependent manner. And as shown in Figure 6B, the three subclusters of cancer types with distinct enrichment patterns in the integrinScore-high group highlight intrinsic differences in the integrin interactomes among different cancers.

### Integrins remodel TME and predict immunotherapy response

**Figure 7:**
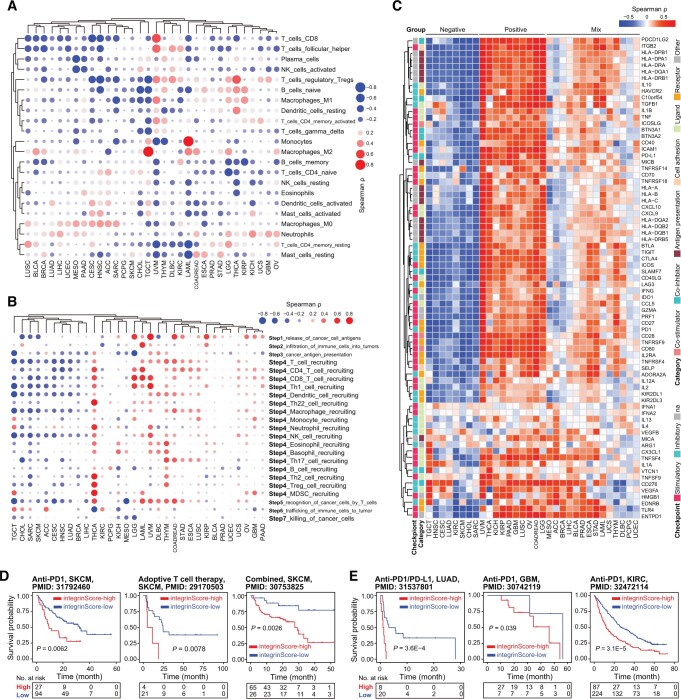
IntegrinScore is correlated with immune features in cancers **A**. Spearman correlation of integrinScore with relative abundance of different types of immune cells across cancers. **B**. Spearman correlation of integrinScore with relative activity of seven immune steps across cancers. Notably, step4 involves various kinds of cells and is shown by cell types. In (A) and (B), blue and red dots denote positive and negative correlations, respectively. The size of dots indicates the absolute Spearman ρ. **C**. Heatmap showing Spearman correlation of integrinScore with ICB markers across cancers. Red and blue colors denote positive and negative correlations, respectively. Distinct types of ICB markers are labeled with different colors. Three subgroups are identified by the unsupervised clustering method. **D**. and **E**. Kaplan–Meier plots showing that integrinScore can predict patient survival after immune therapies in SKCM (D) and three other cancers (E). ICB, immune checkpoint blockade; NK, natural killer; na, not applicable.

Immune-related signatures were repeatedly and differentially enriched in tumors stratified by integrinScore across cancers (Figure 6A and B). To search for details, we performed the following analyses. First, we utilized the Estimation of Stromal and Immune cells in Malignant Tumor tissues using Expression data (ESTIMATE) algorithm [49] to calculate the immuneScore (proportion of immune cells), stromalScore (proportion of stromal cells), and ESTIMATEScore (proportion of nontumor components) for all TCGA samples. We found that integrinScore was strongly associated with these three scores in most cancer types (Figure S7A). Among them, LAML (ρ = 0.70) and TGCT (ρ = −0.76) had the most significant positive and negative correlation with integrinScore, respectively (Figure S7A; Table S11), corroborating the positive and negative enrichment of immune signatures in integrinScore-high group in LAML and TGCT, respectively (Figure 6A and B). Next, we utilized the CIBERSORT algorithm to quantify the relative abundance of 22 immune subsets in each tumor sample. As expected, the infiltration levels of different immune cells were significantly correlated with integrinScore, with heterogeneity in both the correlation direction and strength in a tumor-dependent manner (**Figure 7**A, Figure S7B; Table S11). Importantly, integrinScore was generally negatively correlated with antitumor subsets across cancers, including CD8^+^ T cells, activated natural killer (NK) cells, and memory B cells, while positively correlated with immunosuppressive or inactivated subsets, including M2/M0 macrophage and neutrophils (Figure S7B). To further strengthen our results, we analyzed association between integrinScore and the proportion of distinct tumor-infiltrating lymphocytes (TILs) in several PRAD single-cell RNA-seq (scRNA-seq) datasets. After annotating the cell identity for each population based on commonly-used lineage markers, we calculated the averaged integrinScore for each sample by considering all single cells as a whole set. Consistently, a negative correlation was observed between integrinScore and the CD8^+^ T cell abundance in two datasets (Figure S7C) (the *P* value in the GSE141445 dataset was slightly greater than 0.05 due to a small sample size). Expectedly, the correlation between integrinScore and NK proportion was non-significant in the two datasets, in line with the TCGA-PRAD cohort result (Figure S7C). The antitumor immune response is a series of seven stepwise events called the cancer–immunity cycle [50]. Based on the relative activity score of each immune step in the Tracking Tumor Immunophenotype (TIP) database [50], we computed the correlation between integrinScore and immune steps. Overall, integrinScore was significantly correlated with each step in at least four cancers (*e.g.*, step2) (Figure 7B, Figure S7D; Table S11). IntegrinScore-high tumors in 12 cancer types exhibited higher activity in step1 (release of cancer cell antigens) but lower activity in step3 (cancer antigen presentation) in most cancers (Figure S7D), indicating a defect in the antigen presentation process in integrinScore-high groups in these cancers. Importantly, integrinScore was negatively correlated with the activity of step7 (killing of cancer cells) in six cancer types, with no positive correlation being observed in any other cancers (Figure 7B, Figure S7D; Table S11). To further dissect the underlying link between integrinScore and tumor immunity, we computed their correlation with the expression of an array of well-known immunomodulators [33]. In line with heterogeneous associations between integrinScore and immune cell components as well as immune cycle steps, we also found that integrinScore showed different correlations with them across cancers (Figure 7C). Unsupervised clustering based on the results of correlation analysis revealed three patterns emerged (negative-correlated, positive-correlated, and mix-correlated), regardless of the functional category of immunomodulators (Figure 7C). Globally, TGCT, HNSC, SKCM, CHOL, and SARC were included in the negative group, consistent with reversal correlations between integrinScore and immune signatures and immune cycle steps, in these cancers (Figures 6A, 6B, 7B, and 7C). Particularly, a negative correlation was observed between integrinScore and some well-known immunotherapeutic targets (*i.e.*, PD1, PD-L1, and CTLA4) in TGCT, HNSC, CESC, LUAD, KIRC, SKCM, CHOL, and SARC. In support, DEG analysis revealed the down-regulation of PD1, PD-L1, and CTLA4 in integrinScore-high groups in those cancers (Figure S7E).

The intrinsic link between integrinScore and the immune landscape provided a rationale for assessing whether integrinScore can serve as a tool to predict patients’ response to ICB therapy. In three independent SKCM cohorts treated with anti-PD1 [51], adoptive T cell therapy [52], or combined therapy [53], patients were stratified by integrinScore, and we found that patients in the integrinScore-low group had significantly longer survival time (Figure 7D) and more beneficial responses to ICB. The percentage of responders in integrinScore-high group was 33.3% *vs*. 42.6%, 0 *vs*. 47.6%, and 44.6% *vs*. 76.9% compared with that in integrinScore-low group in anti-PD1, adoptive T cell, and combined therapy cohort, respectively (Figure S7F). Similar findings were observed in other three independent cohorts: a LUAD cohort treated with anti-PD1/PD-L1 (*n* = 28) (Figure 7E, Figure S7G) [54], a GBM cohort treated with anti-PD1 (*n* = 34) (Figure 7E) [55], and a KIRC cohort treated with anti-PD1 (*n* = 311) (Figure 7E) [56]. Although the differences in response rates between integrinScore-high and integrinScore-low groups were not statistically significant in some cohorts owing to a limited number of cases, the rate of beneficial response in the integrinScore-low group consistently tended to be higher in these cohorts (Figure S7F and G). Collectively, these results suggest that integrinScore, which integrates the interplay between integrins and the immune environment, is a potential biomarker for predicting the response of patients to various immunotherapies.

### Identification of compounds potentially targeting the integrin family

**Figure 8:**
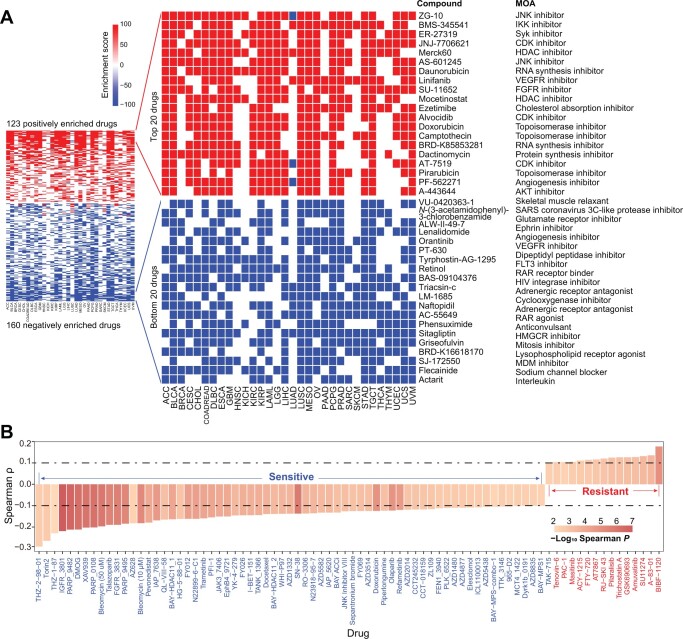
Correlation of integrinScore with drugs **A**. Heatmaps showing the enrichment scores of all 283 significantly enriched (left) and extreme 40 (middle) compounds against integrinScore across cancers by the CMap analysis. Red and blue colors denote positive and negative enrichment, respectively. MOA for each compound is listed on the right. **B**. Spearman correlation of integrinScore with IC50 of different compounds reported in the GDSC database. Pairwise results are filtered by absolute Spearman ρ > 0.1 and *P* < 0.05. Compounds showing positive and negative correlations with integrinScore are considered as resistant (red) and sensitive (blue) drugs. CMap, Connectivity Map; GDSC, Genomics of Drug Sensitivity in Cancer; MOA, mechanism of action; IC50, half maximal inhibitory concentration.

We next employed the CMap resource to identify candidate compounds targeting key signaling pathways intertwined with the integrin family. CMap uses gene expression profiles to uncover associations among genes, chemicals, and biological perturbations [57]. By a stringent cutoff as having a positive or negative correlation with integrinScore in at least 10 cancer types, we identified a total of 283 compounds, with 123 and 160 compounds positively and negatively correlated with integrinScore, respectively (**Figure 8**A; Table S12). These compounds were found to share 340 mechanisms of action (MOAs) (one compound may have multiple MOAs) (Table S12). Topoisomerase inhibitor, cyclin-dependent kinase (CDK) inhibitor, and histone deacetylase (HDAC) inhibitor represented the top 3 shared MOAs (Figure S8A). Among the top 20 positively correlated compounds, several compounds were TME-related, including linifanib [a vascular endothelial growth factor receptor (VEGFR) inhibitor], SU-11652 [a fibroblast growth factor receptor (FGFR) inhibitor], and PF-562271 (an angiogenesis inhibitor). Notably, protein kinase B (AKT) is downstream of integrin activation [4], and we observed that A-443644 (an AKT inhibitor) was positively correlated with integrinScore. These results collectively indicate that targeting tumor TME-remodeling and aggressiveness-promoting pathways represents a therapeutic strategy for cancers driven by overrepresentation of integrin genes. Among the top 20 negatively correlated compounds, some compounds were metabolism-related, including ALW-II-49-7 (a glutamate receptor inhibitor), tyrphostin AG-1295 (a dipeptidyl peptidase inhibitor), and sitagliptin [a 3-hydroxy-3-methylglutaryl-CoA reductase Gene (HMGCR) inhibitor], implying that they could be used to modulate integrin expression. Targeted gene analysis uncovered 480 unique drug-targeted genes shared by the abovementioned compounds, with *TOP2A*, *CHRM1/2/3/4*, and *CDK1/2* being the top ones (Figure S8B; Table S12).

Alternatively, utilizing the GDSC database [58], we identified 80 significant correlations between integrinScore and the half maximal inhibitory concentration (IC50) of drugs (Figure 8B; Table S13). Specifically, the IC50 values of 15 drugs were positively correlated with integrinScore (indicating drug resistance), whereas those of 65 drugs were negatively correlated with integrinScore (indicating drug sensitivity), suggesting potential therapeutic strategies to treat integrinScore-high tumors with these sensitive drugs. Targeted pathway and gene analyses revealed that genome integrity, kinases, and receptor tyrosine kinase (RTK) signaling, and *PARP1/2* and *IAP* represented the most affected pathways (Figure S8C) and genes (Figure S8D), respectively. Phosphoinositide 3-kinase (PI3K), mitogen-activated protein kinase (MAPK), and extracellular regulated protein kinase (ERK) are downstream of integrin activation [4], and we observed that Torin2 and AZD8835 (both targeting PI3K) as well as AZ628 and refametinib (both targeting ERK/MAPK) were negatively correlated with integrinScore, implying an antagonistic role of these compounds against integrin signaling. Notably, 33.3% of targeted genes identified from GDSC overlapped with those from CMap (Figure S8E), providing an array of actionable targets for interfering with aberrant integrin activity.

To further biologically testing these compounds, we selected Torin2 (a potent and selective ATP-competitive mTOR inhibitor) and XAV939 (a Wnt/β-catenin inhibitor) for experimental validation. Using AR^+^ LNCaP cells as a prostate cancer (PCa) model, Torin2 (Figure S8F) and XAV939 (Figure S8G) treatment for 3 days disturbed the expression of 10 and 6 out of 12 integrin genes, respectively. These results strongly suggest that our identified drugs can modulate the expression pattern of integrin family. Consequently, treatment with the two drugs significantly inhibited the proliferation of PCa cells, with Torin2 being more toxic (Figure S8H). Next, Western blot was performed to evaluate the activity of mitotic pathways (*i.e.*, PI3K/AKT, ERK, and p38 MAPK) downstream of integrin signaling. Results showed that not only the total protein level but also the activated phosphorylated form of these pathways were markedly suppressed by Torin2 (Figure S8I). Collectively, we identified several compounds potentially altering integrin gene expression, which thus may constitute alternative strategies to targeting cancers harboring aberrant integrin activities.

### Construction of a user-friendly integrin database

**Figure 9:**
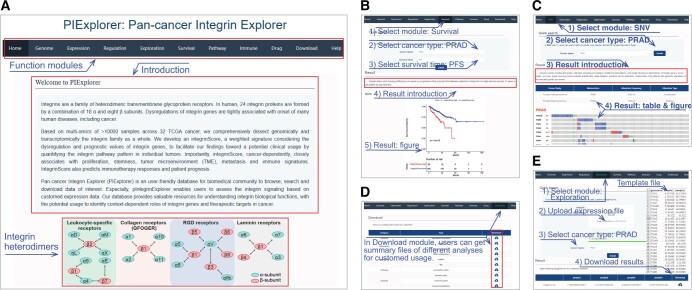
Screenshots for PIExplore database usage **A**. Screenshots for the homepage of PIExplore website. **B**.–**E**. Usage instructions for “Survival”, “SNV”, “Download”, and “Exploration” modules. Red boxes and blue lines indicate highlighted contents and explanations, respectively. SNV, single nucleotide variant; PIExplore, Pan-cancer Integrin Explorer.

To publicly share our findings, we developed an open resource tool called PIExplorer (http://computationalbiology.cn/PIExplorer). Our tool includes nine functional modules that allow worldwide users to browse and download the majority of integrin-related data presented in this study (**Figure 9**A). After the users submit their requests, the results are displayed as tables in all modules, except for “Survival”, “Genome”, and “Expression” modules, in which figures are shown and available for download directly (Figure 9B and C). All tables obtained from distinct modules are accessible in the “Download” function (Figure 9D). Particularly, we built an “Exploration” module, enabling users to calculate integrinScore for their own samples based on their transcriptomic data (Figure 9E). Therefore, our PIExplorer is a valuable resource for biomedical community to systematically conduct integrin gene analysis across human cancer types.

## Discussion

Integrins are heterodimeric transmembrane receptors that regulate cellular growth, proliferation, and migration by transmitting bidirectional biochemical signals between cells [1,4,8]. Studies at the individual gene level have shown that integrins are involved in tumorigenesis; however, a comprehensive characterization of the integrin family and associated signatures at the pan-cancer level is lacking. Here, we interrogated TCGA data, including genomic alterations, transcriptomic abnormalities (as well as the underlying molecular mechanisms such as genetic and epigenetic regulation), and clinical outcomes, to systematically profile the integrin pathway in nearly 10,000 tumors spanning 32 cancer types and validated the results in multiple external cohorts. The results reveal not only extensive and context-dependent dysregulation patterns of integrin genes but also their cancer-specific interactomes across distinct cancers, providing a global picture of the functional heterogeneity of integrins in human cancers.

Although integrins display a very low alteration frequency at individual gene level, they constitute collectively a frequently altered pathway at the population level, indicating involvement in tumorigenesis. In support, studies have shown that the T188I mutation in ITGB1 contributed to neoplasia by over-activating ERK/MAPK signaling [59], and mutations in *ITGA2* were associated with an increased risk of prostate, oral, and breast cancers [60]. Interestingly, we observed a sexual dimorphism in integrins alteration among female-specific and male-specific cancers, in that the amplification frequency in female cancers is significantly higher than that in male cancers (Figure 1C). These findings suggest a potential relationship between integrins and sex hormones and provide a rationale for considering gender difference in applying integrin-targeted therapies in these particular cancer types. Consistently, studies have shown that estrogen or androgen can directly bind to the regulatory elements of integrin genes to modulate their expression, causing changes in cancer cell behaviors [61–64].

Compared to the relatively limited number of genomic alterations, transcriptional dysregulation of integrins genes is much more predominant. Changes in the expression of certain integrins have been documented to be linked to tumorigenesis in multiple organs [4,6,65]. Here, we found that aberrant expression of most integrin genes was associated with patient outcomes in a context-dependent manner (Figure 4D, Figure S4). For example, in terms of prognosis analysis, certain integrins may play a protective role in one cancer but an oncogenic role in another cancer. In given cancer types, the majority of dysregulated integrins may function as tumor suppressors or promoters. Therefore, our findings highlight an intricate landscape of functional heterogeneity of integrin genes among distinct cancers, which should be considered when develop and apply integrin-targeted therapies.

Misexpression, but not the genomic alterations, of integrin genes, successfully classified patient cohorts into four clusters exhibiting distinct molecular and clinical features (Figures 2 and 4). By analyzing genetic (*i.e.*, CNV and TFs) and epigenetic (*i.e.*, DNA methylation and miRNA) mechanisms, we showed that dysregulation of integrin genes in cancer is regulated at versatile levels that constitute a sophisticated regulatory network, expanding our understanding of integrin pathway. Considering the prognostic values and the expression patterns of dysregulated integrin genes in a given cancer type, we constructed integrinScore to measure an overall integrin signaling pattern in individual tumor samples, with an aim to translate such integrin dysregulation-driven biology toward a potential clinical use. The integrinScore separated each cancer cohort into two groups, with the integrinScore-high group being more aggressive. Importantly, integrinScore was tightly correlated with numerous predefined genomic, transcriptomic, epigenetic, and clinical subtypes, demonstrating its utility and generality. Molecularly, integrinScore was cancer-dependently associated with cancer hallmarks and other signatures, including proliferation, stemness, TME/metastasis, and immune features (Figures 6 and **7**). Generally, tumors originating from the same tissue usually have similar gene expression and regulatory networks [37,66]. To our surprise, we found that the patterns of integrin expression and interactome were obviously different among KIRC, KIRP, and KICH (Figures 2A, 6A, and 6B). Although few reports have revealed detailed molecular differences among these three kidney cancers, they do differ in biology [67]. Our data imply that, compared to other well-studied oncogenic pathways, integrin signaling may represent a better biomarker to distinguish these three kidney cancers.

Metastasis is the leading cause of cancer-related death. Here, we surprisingly found that integrinScores were overwhelmingly higher in metastatic *vs*. primary tumors, consistent with the intrinsic role of integrins in modulating cell motility via downstream functional signaling such as focal adhesion kinase (FAK), Src family kinase (SFK), and PI3K. Metastatic tumors are usually treatment-resistant. Based on the close relationship between TME and integrinScore, we demonstrated that integrinScore can be used to predict patient’s response and outcome to ICB. This indicates that a combination of ICB with an integrin-targeted regimen may be beneficial. Consistently, clinical trials evaluating the efficacy of this combination therapy are underway [1,4,5]. Furthermore, numerous compounds that potentially interfere with integrin signaling were identified using the CMap and GDSC databases. Many compounds were found to target the downstream effectors of integrins (*e.g.*, AKT1/2/3, FGFR1/2/3, and mTOR). In addition, we provided experimental evidence that some of the top compounds affected the expression of integrin genes and proliferation in cancer cells, offering a new avenue of combining these potential drugs with ICB to achieve better therapeutic outcomes [4].

Collectively, our findings highlight the integrin family as a potential vulnerability for treating aggressive tumors. To facilitate the spreading of our findings, we built an interactive website named PIExplorer to allow the biomedical community to access the data of interest and evaluate integrin signaling using their data. However, there are limitations to our study. First, our results are mainly based on *in silico* analysis, so further large-scale experimental validations in different cancer-specific contexts are needed. It is noteworthy that we validated our results in several non-TCGA datasets, indicative of the validity of our analytical pipeline. Second, although integrinScore efficiently stratify and predict patients’ immunotherapy responses at the population level, the model may suffer limitation when applied to individual patients or a small cohort. This limitation can be reconciled by the integrated use of integrinScore and other biomarkers to render an accurate stratification. Third, integrin proteins function through heterodimers, our current scoring model does not consider the pairing owing to an extreme expression heterogeneity among integrin genes. An algorithm that considers heterodimer combinations based on the transcriptome of 26 integrin genes is expected to further improve the statistical ability of our model. Fourth, due to the limited studies of immunotherapies across cancers, we only observe an association between integrinScore and response/outcome in several cohorts (mainly SKCM), clinical application of integrinScore in other cancer types requires more investigation.

## Materials and methods

### Datasets and cohorts

Expression data of mRNA and miRNA, DNA methylation data, and clinical data for TCGA samples were downloaded from the University of California Santa Cruz (UCSC) Xena (https://xenabrowser.net/). Mutation data for TCGA samples were collected from cBioPortal [16]. Expression profiles for validation of the correlation between integrin genes and TFs (Figure S3K and L) were downloaded from the Gene Expression Omnibus (GEO: GSE157548) and the CGGA database (http://www.cgga.org.cn/). Transcriptomic and clinical data for validation of integrinScore prognostic value (Figure S5B) were downloaded from the International Cancer Genome Consortium (ICGC) Data Portal (https://dcc.icgc.org/). Expression data for metastatic cancer were collected from the MET500 cohort (https://met500.med.umich.edu/). Expression and clinical data for immunotherapy were downloaded from GEO or collected from the Tumor Immunotherapy Gene Expression Resource portal (http://tiger.canceromics.org/). Known subtypes or classifications of human cancers were collected from original publications as described in the Results section.

### Integrin gene expression analysis

We obtained the normalized gene expression data [count and transcripts per kilobase of exon model per million mapped reads (TPM)] for TCGA samples from the UCSC Xena. For DEG analysis, gene counts for samples were first classified into different groups, then the DESeq2 package [68] was utilized to estimate fold change (FC) of each gene, and genes with absolute FC ≥ 2 and false discovery rate (FDR) < 0.05 were identified as DEGs. For other analysis, including multiple association analysis, survival analysis, and unsupervised clustering analysis, gene TPM values were utilized (Figures 2C, 3A, 3D, 3H, 3K, 4B, 4D, 4E, and 4G, Figures S2A–C, S2E–H, S3A–D, S3G–I, S3L–M, and S4).

### DNA methylation analysis

The Illumina Infinium Human Methylation 450K for 9639 tumor samples was used for analysis. We mapped all probes to individual genes according to their location on the human genome. Gene promoter was defined as the genomic interval from 1000 bp upstream to 200 bp downstream of the transcription start site. Only probes overlapped with integrin genes promoter were retained for further analysis. For multiple probes overlapping with the same integrin gene, the probe with the highest average β-value was used for the final analysis. Significant correlations were filtered by absolute Spearman ρ ≥ 0.15 and *P* < 0.05.

### miRNA and TF analyses

For miRNA analysis, we first collected miRNAs with available expression data for TCGA samples and subsequently annotated them by starBase [69] and miRTarBase [70]. To remove miRNAs with extremely low expression, only miRNAs with normalized expression > 1 in more than 500 samples were retained. The correlation between miRNA and integrin genes was calculated by the Spearman method and pairs with absolute ρ ≥ 0.3 and *P* < 0.05 in more than five cancer types were regarded as significantly correlated. To enhance data quality, 40 significant miRNA–integrin pairs with validation in starBase or miRTarBase were used for further analysis.

For TF analysis, we first downloaded all TF–target pairs, including 495 TFs and 38,183 targets, from the hTFtarget database (http://guolab.wchscu.cn/hTFtarget/#!/). The correlation between TFs and target genes was calculated by Spearman method and pairs with absolute ρ ≥ 0.2 and *P* < 0.01 were retained for further analysis. The top 10 TFs with the most integrin target genes were shown (Figure 3J). For integrated analysis of miRNAs and TFs (Figure S3N), we first extracted 27 miRNAs and 11 integrins in the 40 miRNA–integrin pairs (Figure 3H). Then, 21 TFs targeting all 11 integrins were identified from the hTFtarget database. Next, associations between the 27 miRNAs and 21 TFs were calculated by miRTarBase. Finally, a reciprocally regulated network between miRNAs, TFs, and integrins was constructed by Cytoscape software.

### Survival analysis

The survival differences between different groups [*e.g.*, patients with/without integrin gene alteration (Figure S1B–E), and patients in different clusters (Figure 2B)] were estimated by Kaplan–Meier curve, and *P* < 0.05 was considered as statistically significant. For continuous variables like gene expression or integrinScore, the “sur_vcutpoint” function of “survminer” package was first applied to determine the optimal cutoff for maximum rank statistic, and then the “survival” R package was utilized to compare survival differences between high and low groups.

### IntegrinScore computation

We computed integrinScore for each sample in a way used in our recent study [38]. Briefly, we first performed survival analysis for integrin genes across 32 cancer types. We utilized two kinds of outcomes (OS and PFS) in a cancer-dependent manner for survival analysis, as recommended previously [71]. The oncogenic integrin gene was identified as when patients had higher gene expression, their survival time was significantly shorter; while the protective integrin gene was identified by the opposite trend. Then, the expression levels of 26 integrin genes for each sample were extracted and normalized. And the integrinScore was calculated based on a linear combination of normalized expression values of the 26 integrin genes, of which oncogenic and protective integrin genes contributed positively and negatively to integrinScore, respectively:

integrinScore=Σ(oncogenic integrin genes)-Σ(protective integrin genes)

### Biological pathway/signature analysis

We identified biological pathways/signatures associated with integrinScore by the pre-ranked module embedded in GSEA [72]. After stratifying patients into integrinScore-high and integrinScore-low groups, DEGs were identified and all genes were ranked by FC. Then, hallmark gene sets and curated signatures (mainly from C2-all which contained 7233 gene sets) downloaded from the GSEA website (MSigDB; https://www.gsea-msigdb.org/gsea/msigdb) were enriched against ranked gene lists by clusterProfiler package [73], and signatures with FDR < 0.1 were considered statistically significant. For the proliferation-related signatures in Figure 4B, the relative activity score of four signatures was estimated by GSVA package [36] in the R environment.

### Immune cell, cycle, and modulator analyses

The ESTIMATE algorithm was used to score for tumor purity, the level of stromal cells present, and immune cell infiltration in tumor tissues based on expression data [49]. The CIBERSORT is a deconvolution tool to estimate the abundances of immune cell types in a mixed cell population using gene expression data [74]. We downloaded CIBERSORT results for TCGA pan-cancer samples from the Genomic Data Commons (GDC) PanCanAtlas (https://gdc.cancer.gov/about-data/publications/panimmune). A previous study has conceptualized the anticancer immune response as seven sequential steps: (1) release of cancer cell antigens; (2) cancer antigen presentation; (3) priming and activation; (4) trafficking of immune cells to tumors; (5) infiltration of immune cells into tumors; (6) recognition of cancer cells by T cells; and (7) killing of cancer cells. In aggregate, these seven steps were referred to as the cancer immunity cycle [50]. We downloaded the relative activity score of seven steps for TCGA pan-cancer samples from the TIP portal (http://biocc.hrbmu.edu.cn/TIP/). An array of immunomodulators were obtained from the TCGA immune response working group [33]. Spearman ρ values between integrinScore and immune cells/cycles/modulators were calculated for distinct cancers.

### Drug analysis

For CMap analysis, we first performed DEG analysis for samples stratified by integrinScore of each cancer type. Then the top 150 up-regulated genes (integrinScore-high group *vs*. integrinScore-low group) were subjected to the query module of the CMap online tool (https://clue.io/). Compounds with enrichment scores > 95 or < −95 in at least ten cancer types were considered as significantly positive or negative compounds, respectively. For GDSC analysis, we first calculated integrinScore for each cell line according to distinct cancer types. Then, the Spearman ρ between integrinScore and IC50 of different drugs was calculated, and pairs with absolute ρ ≥ 0.1 and *P* < 0.05 were considered significantly correlated.

### Drug treatment, methyl thiazolyl tetrazolium assay, quantitative reverse transcription-polymerase chain reaction, and Western blot analysis

For all drug treatment experiments, drugs were dissolved in dimethyl sulfoxide (DMSO) separately. The effects of single drugs were pre-determined and doses lower than IC50 were used. Torin2 (Catalog No. T6100, TargetMol, Shanghai, China), 5′-Aza (Catalog No. T1339, TargetMol), enzalutamide (Catalog No. T6002, TargetMol), and XAV939 (Catalog No. T1878, TargetMol) were purchased from TOPSCIENCE. For methyl thiazolyl tetrazolium (MTT) assays, cells were trypsinized, counted, and seeded in 96-well plates, and then, after drug treatment for 3–4 days, MTT was added at a concentration of 0.5 mg/ml for 3 h at 37°C. The medium was then removed and 0.2 ml/well of acidic isopropyl alcohol (0.04 M HCl in absolute isopropyl alcohol) was added. The absorbance of the converted dye was measured at 570 nm using a Synergy 2 Spectrophotometer (BioTek, VT). Quantitative reverse transcription-polymerase chain reaction (qRT-PCR) was performed using the iQ SYBR Green Supermix (Bio-Rad, CA) on a 7900HT Fast Real-Time PCR System (Applied Biosystems, CA). The primers used in this study are listed in Table S14. Normally, *GAPDH* or *ACTB* was used as internal control for gene expression normalization. For Western blot analysis, antibodies against p-AKT, AKT1/2/3, p-ERK1/2, ERK1/2, p-p38, p38, and β-tubulin were used (Table S14).

### Database construction

We constructed the PIExplorer database by the standard Model–View–Controller (MVC) pattern, which consists of a server side and a client side. The frontend was built by Vue and Element UI. The backend was encoded by Java, and raw data were stored and managed by the MySQL, with Spring Boot, MybatisPlus, and Redis providing interface services. The outputs of database are tables and/or figures.

### Statistical analysis

All statistical analyses in our study were performed by R 4.1.0. Specifically, analysis of DNA methylation, miRNAs, and TFs, as well as the association between integrinScore and selected signatures, cancer immunity cycle, 22 immune cell subpopulations, and marker genes, were performed by Spearman analysis (Figures 3D, 3H, 3K, and 7A–C, Figures S5C, S6D, and S7A). Quantitative data fitting normal distribution was compared by *t*-test; otherwise, the Wilcoxon test (for two groups) or Kruskal–Wallis test (for more than two groups) was used. Chi-square test or Fisher’s exact test was performed to compare differences between categorical variables. Heatmaps showing Spearman ρ, percentage, FC, and *P* value (Figures 2A, 2C, 3D, 3H, 3K, 4D, 6A, 6B, 7A, and 7B, Figures S2H, S4A, S6C, S6D, and S7A) were visualized by TBtools [75].

## CRediT author statement

**Cheng Zou:** Conceptualization, Methodology, Software, Validation, Formal analysis, Investigation, Data curation, Writing – original draft, Visualization, Funding acquisition. **Jinwei Zhu:** Methodology, Software, Formal analysis, Investigation, Data curation. **Jiangling Xiong:** Investigation, Data curation, Validation. **Yu Tian:** Validation. **Yousong Peng:** Methodology, Software, Resources. **Edwin Cheung:** Methodology, Software, Resources. **Dingxiao Zhang:** Conceptualization, Methodology, Writing – original draft, Writing – review & editing, Visualization, Supervision, Project administration, Funding acquisition. All authors have read and approved the final manuscript.

## Supplementary material

Supplementary material is available at *Genomics, Proteomics & Bioinformatics* online (https://doi.org/10.1093/gpbjnl/qzae035).

## Competing interests

The authors have declared no competing interests.

## Supplementary Material

qzae035_Supplementary_Data
