# Supplementary material for: Comprehensive Characterization of the Integrin Family Across 32 Cancer Types
Source: Genomics Proteomics Bioinformatics. 2024 May 9;22(4):qzae035. doi: 10.1093/gpbjnl/qzae035 (PMC11849494; doi:10.1093/gpbjnl/qzae035)
Supplement: qzae035_Supplementary_Data [file qzae035_supplementary_data.zip › Supplementary material captions.docx]

**Supplementary material**

**Figure S1 Integrin alterations rarely correlate with patient’s survival time**

**A.** Distribution of mutation, amplification, and deletion frequencies of 26 integrin genes across cancers. Data shown in the panel are frequency values. **B.** and **C.** Kaplan–Meier plots show that integrin alterations are associated with shorter OS (B) and PFS (C) time. **D.** and **E.** Summary of survival analysis results (OS in D and PFS in E) of integrin genes across cancers. Red, blue, and black dots indicate worse, better, and unsignificant results, respectively. Alteration groups with < 5 samples are not performed survival analysis and denoted as NA (grey dots). The size of dots indicates −log_2_(*P* values). Data in the parentheses (top left) denote the rate of alterations associated with survival.

**Figure S2 Integrin general expression patterns correlate with patient’s outcome**

**A.** Unsupervised clustering based on the expression of 26 integrin genes showing k = 4 (red point) as the optimal consensus clusters. **B, C, E,** and **F.** Integrin general expression patterns consistently correlates with OS (B and C) and PFS (E and F) when k = 3 (B and E) and k = 5 (C and F), respectively. **D.** and **G.** Venn plot showing integrin general expression can convergently identify a subgroup of tumors with worse outcomes (OS and PFS for D and G, respectively). **H.** The expression repertoire of 26 integrin genes classifies TCGA tumors into four clusters (termed clu_1/2/3/4; left). Boxplot (right) showing difference in expression of 26 integrin genes among four clusters. Significance is calculated by the Kruskal–Wallis test. Within the boxplots, the center lines represent median values, box edges are 75th and 25th percentiles, and dots denote the outliers. CDF, cumulative distribution function.

**Figure S3 Multiple mechanisms combinedly regulate integrin expression**

**A.** Dot plots showing Spearman correlation between DNA methylation in promoter and expression of integrin genes. Two integrin genes in two cancers are shown. **B.** and **C.** Inconsistency between expression and methylation level for *ITGA11* in LIHC (B) and *ITGB7* in THCA (C). Top and middle are the violin plots showing DNA methylation in promoter and expression of integrin gene in tumors and normal tissues, respectively; at the bottom are dot plots showing Spearman correlation between DNA methylation in promoter and expression of integrin genes. Within the violin plots, the center white dots represent mean values, box edges are 75th and 25th percentiles, and dots denote the outliers. **D.** Density plots showing correlation coefficient between 40 pairs of miRNAs and integrin genes in TCGA tumors. The median is labeled. **E**. Validation of the correlation between miRNA and integrin gene in TCGA (top panel) and independent liver cancer cohort (bottom panel). **F.** Bar plots showing the number of significant correlated miRNA–integrin pairs across cancers. The cutoff of Spearman ρ ≥ 0.3 and *P* < 0.05 is considered significant. **G.–I.** Correlation networks of miRNA–integrin pairs in COADREAD (**G**) and LGG (H), and miRNA–targets pairs (I). The same miRNA–integrin pairs have predominantly negative correlations in COADREAD but positive correlations in LGG. Two miRNAs with positive correlation with part of their targets in LGG are shown as examples. The size of circle or V-shape plots denotes the number of pairs directly associate with this gene. **J.** Alluvial plot showing the top ten TFs with most integrin targets. Numbers in the parentheses are the number of integrin targets. **K.** Bar plots showing fold change of *FOXA1* and its integrin targets in PRAD and HNSC tumors compared with normal tissues. *P* values are labeled. **L.** and **M.** The correlation between TFs and integrin genes are validated in different cohorts (L for PRAD; M for LGG). Dot plots show the relationship between individual TF and integrin gene labeled by correlation coefficients and *P* values. **N.** A three-way interacting network between miRNA, TFs, and integrin genes (left). A MYC-centered network is shown as an example (right). Lines indicate regulation relationship. The size of circle, triangle, and V-shape plot denotes the number of pairs directly associate with this gene. Circles, integrin genes; triangle, TFs; V-shape plots: miRNAs. **O.–Q**. qPCR analysis of 12 integrin gene expression in prostate cancer LNCaP cells treated with 5'-Aza (O), miR-92a-3p (P), and Enza (Q). The cutoff for significance is FC > 1.5 and *P* < 0.05. qPCR, quantitative polymerase chain reaction; DMSO, dimethyl sulfoxide; NC, negative control.

**Figure S4 Heterogeneity of clinical relevance of integrin genes in a cancer-depend manner**

**A.** Summary of PFS time analysis results of integrin genes across cancers. Red, blue, and grey dots indicate worse, better, and unsignificant results, respectively. The size of dots indicates −log_2_(*P* values). Data in the parentheses (top left) denotes the rate of dysregulation associated with PFS. **B.–D.** Three examples (B for *ITGA5*, C for *ITGB7*, and D for *ITGAE*) of cancer-specific associations between indicated integrin gene expression and OS.

**Figure S5 IntegrinScore correlates with outcomes and known molecular subtypes in distinct cancers**

**A.** Summary of OS and PFS time analysis results of integrinScore across cancers. Red and grey dots indicate worse and unsignificant results, respectively. The size of dots indicates −log_2_(*P* values). **B.** Kaplan–Meier plots showing that integrinScore consistently correlates with worse outcome in distinct cohorts. **C.** Spearman correlation of integrinScore with two proliferation related signatures. Blue, red, black, and grey dots denote positive, negative, and unsignificant correlations, and absent values, respectively. The size of dots indicates the absolute correlation coefficient. **D.–F.** Left is violin plots showing integrinScore among four TME subtypes in ACC (D), OV (E), and LGG (F); right is Kaplan–Meier plots showing that integrinScore combined with known subtypes can further identify more aggressive tumors. **G.** Left is an overview of the association between known molecular subtypes and integrinScore in GBM. Middle and right are violin plots showing integrinScore among distinct molecular subtypes. **H.** Left and middle are violin plots showing integrinScore among distinct molecular subtypes in LUSC. Right is comparison of the percentage of four TME subtypes among distinct subgroups of PARADIGM, miRNA, and MDSC. **I.** Comparison of the percentage of four TME subtypes among subgroups in PAAD. **J.–W.** An overview of the association between known molecular subtypes and integrinScore across in BLCA (J), TGCT (K), THCA (L), COADREAD (M), KIRC (N), KIRP (O), LIHC (P), MESO (Q), OV (R), SKCM (S), STAD (T), BRCA (U), CESC (V), and UCEC (W). For example, in THCA, tumors resembled the BRAF-like mutant profile had lower differentiation score and poor outcomes (PMID: 25417114). Consistently, the BRAF-like tumors had higher integrinScore in our study (L). Besides, Berger et al. utilized 16 combined features to category 2579 gynecological (OV, UCEC, CESC, and UCS) tumors into 5 clusters (C1–C5), and OV tumors were mainly classified into C4 (225/418), in which the tumors had the poorest prognosis (PMID: 29622464). As expect, the C4 tumors showed higher integrinScore than that in other clusters (R).

**Figure S6 IntegrinScore correlates with stemness and immune markers in cancer**

**A.** Bar plots showing the number of DEGs between integrinScore high and low groups across cancers. **B.** Bar plots showing the number of cancer types in which each signature significantly enriches. Four types of signatures are labeled with different colors. **C.** Heatmap showing FCs of stemness markers between integrinScore high and low groups across cancers. Red and bule colors denote up-regulation and down-regulation of genes. FCs are labeled in boxes. **D.** Spearman correlation of integrinScore with expression of epithelial and mesenchymal markers. Blue and red dots denote positive and negative correlations, respectively. The size of dots indicates the absolute correlation coefficient. DEGs, differentially expressed genes.

**Figure S7 IntegrinScore predicts patient responses to immune therapy**

**A.** Spearman correlation of integrinScore with immune landscape score across cancers. Blue and red dots denote positive and negative correlations, respectively. The size of dots indicates the absolute correlation coefficient. **B.** Bar plots showing the number of cancer types in which integrinScore significantly correlates with immune cell abundance. The cutoff is labeled on the top. **C.** The correlation between integrinScore and the proportion of CD8^+^ T cell and NK cell in three single-cell prostate cancer datasets. Three PRAD scRNA-seq datasets (GSE181294, GSE141445, and GSE143791) were downloaded from GEO database. After standard quality control, we annotated the cell identity for each subpopulation by commonly-used lineage markers. The integrinScore was deduced based on average transcriptome by treating all single-cells as a whole for a sample. Finally, we calculated the correlation between integrinScore and TIL proportions by Spearman correlation analysis. **D.** Bar plots showing the number of cancer types in which integrinScore significantly correlates with immune steps. The cutoff is labeled on the top. **E.** Heatmap showing FC of three ICB markers between integrinScore high and low groups across cancers. Red and bule colors denote up-regulation and down-regulation of genes. FCs are labeled in boxes. **F.** and **G.** Comparison of the percentage of patients’ response to immune therapies between integrinScore high and low group in SKCM (F) and LUAD (G). Chi-square test is used. TIL, tumor-infiltrating lymphocyte; scRNA-seq, single cell RNA sequence; GEO, Gene Expression Omnibus.

**Figure S8 Pathways and target genes of drugs correlated with integrinScore**

**A.** and **B.** Bar plots showing top MOA (A) and target genes (B) of compounds correlated with integrinScore by the CMap analysis. **C.** and **D.** Bar plots showing top pathways (C) and target genes (D) of compounds correlated with integrinScore in the GDSC database. **E.** Venn plot showing the overlapping of target genes based on the CMap and GDSC database. **F.** and **G.** qPCR analysis of 12 integrin gene expression in LNCaP cells treated with Torin2 (F) and XAV939 (G). The cutoff for significance is fold change > 1.5 and *P* < 0.05. **H.** Cell viability (MTT assay) in LNCaP cells treated with Torin2 and XAV939 for 3–4 days. **I.** Western blot analysis of indicated mitotic pathways in LNCaP cells treated with Torin (50 nM for 3 days). MTT, methyl thiazolyl tetrazolium.

**Table S1 Abbreviation of 32 cancer types in TCGA**

**Table S2 Alteration frequency of 26 integrin genes across 32 cancer types in TCGA**

**Table S3 FC and FDR of 26 integrin genes across 15 cancer types**

**Table S4 Cluster (k = 4) and survival information for TCGA samples**

**Table S5 *P* value and spearman correlation between DNA methylation and expression of integrin genes in pan-cancer**

**Table S6 *P* value and spearman correlation between miRNA and integrin gene expression in pan-cancer**

**Table S7 *P* value and spearman correlation between TFs and integrin gene expression in pan-cancer**

**Table S8 *P* value for analysis of integrin genes and survival time in pan-cancer**

**Table S9 Normalized enrichment score (NES) and FDR of 50 hallmarks analyzed by GSEA across 32 cancer types**

**Table S10 Normalized enrichment score (NES) and FDR of 40 curated signatures analyzed by GSEA across 32 cancer types**

**Table S11 *P* value and spearman correlation between integrinScore and immune features in pan-cancer**

**Table S12 Enrichment score of each compound from the CMap across 32 cancer types**

**Table S13 *P* value and spearman correlation between integrinScore and IC50 of different drugs in GDSC**

**Table S14 List of primers and antibodies used in this study**
