# Supplementary figures and images for: Comprehensive Characterization of the Integrin Family Across 32 Cancer Types
Source: Genomics Proteomics Bioinformatics. 2024 May 9;22(4):qzae035. doi: 10.1093/gpbjnl/qzae035 (PMC11849494; doi:10.1093/gpbjnl/qzae035)

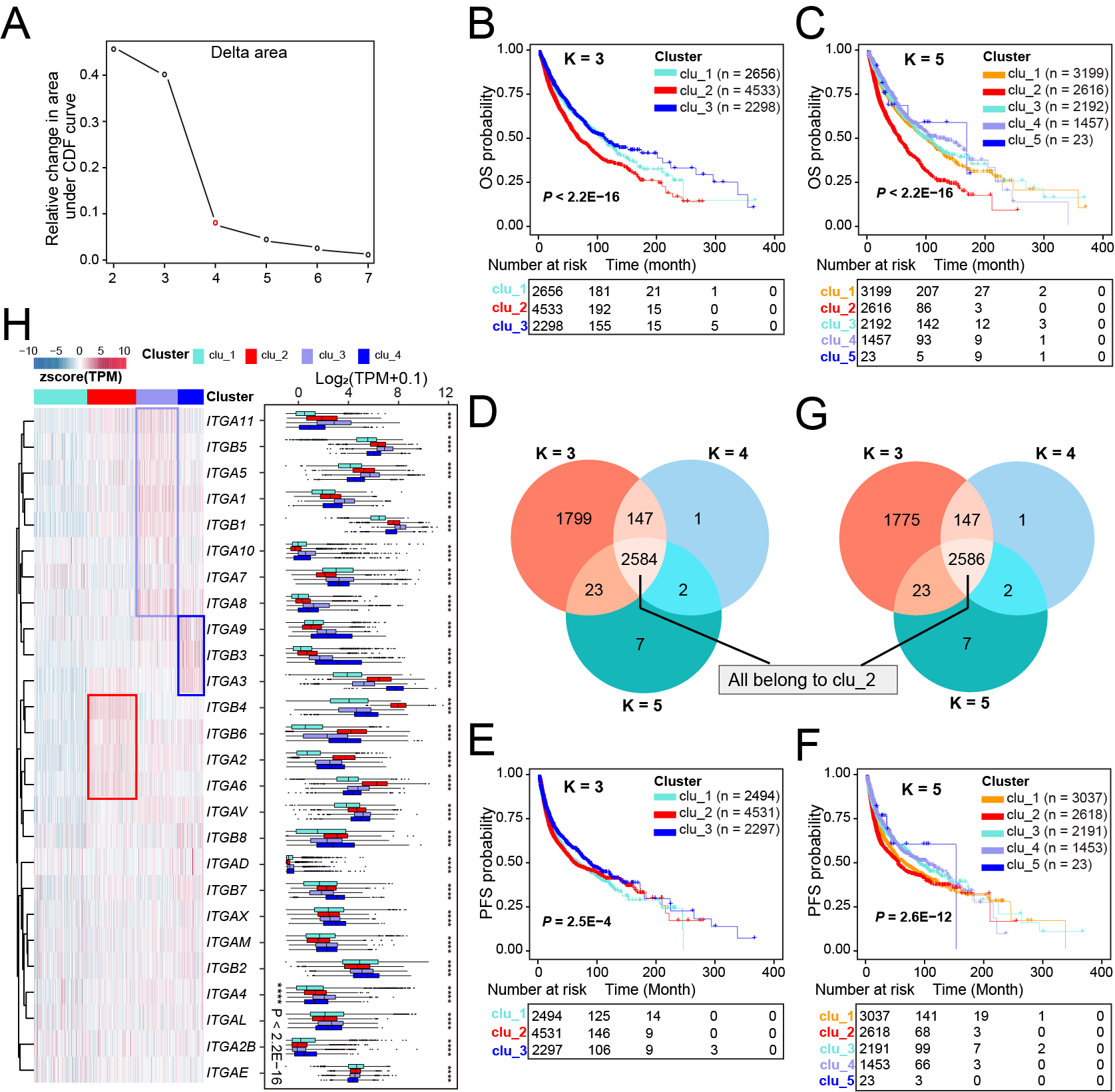

Supplement: qzae035_Supplementary_Data [file qzae035_supplementary_data.zip › Figure_S2.png]

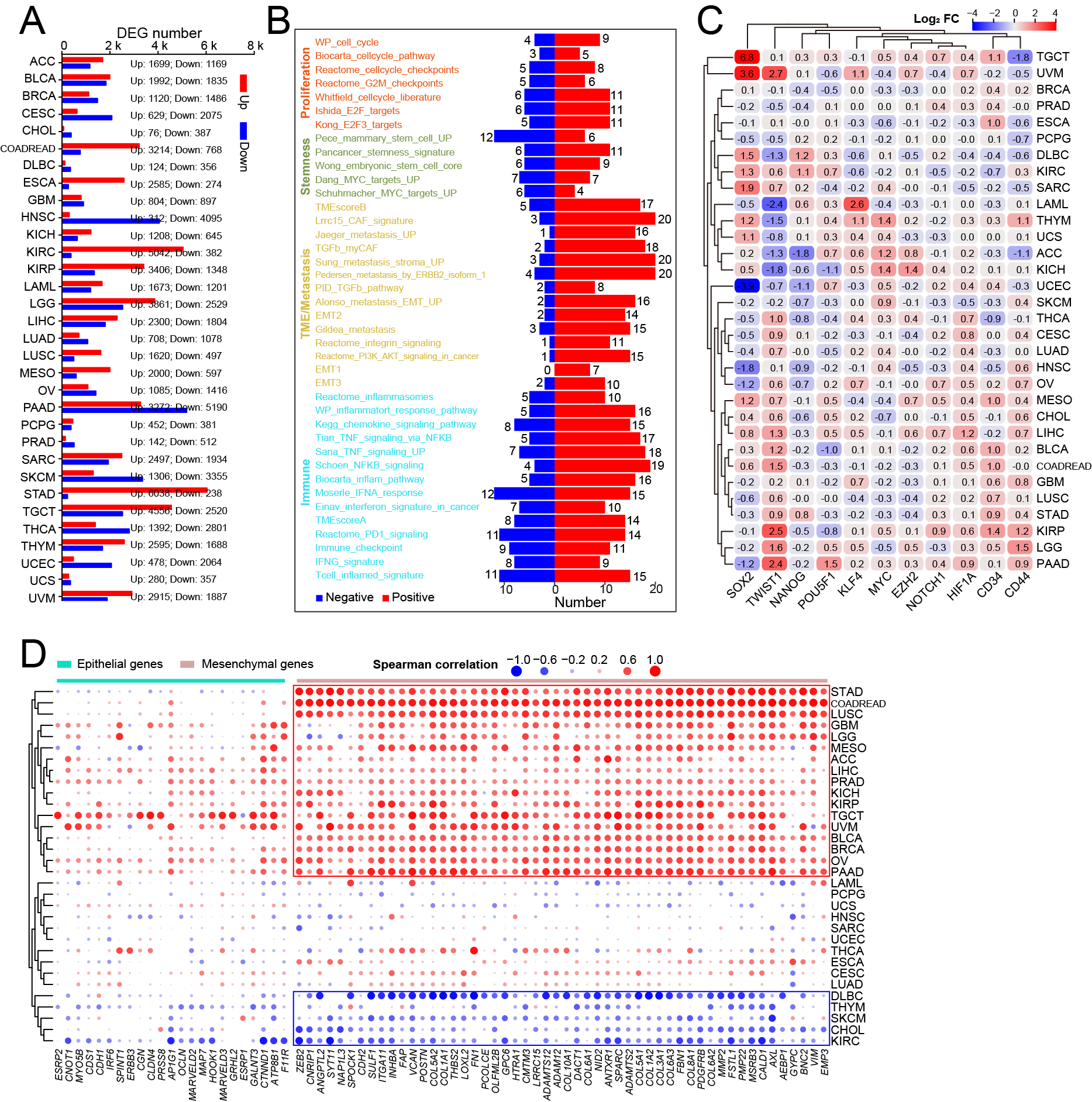

Supplement: qzae035_Supplementary_Data [file qzae035_supplementary_data.zip › Figure_S6.png]

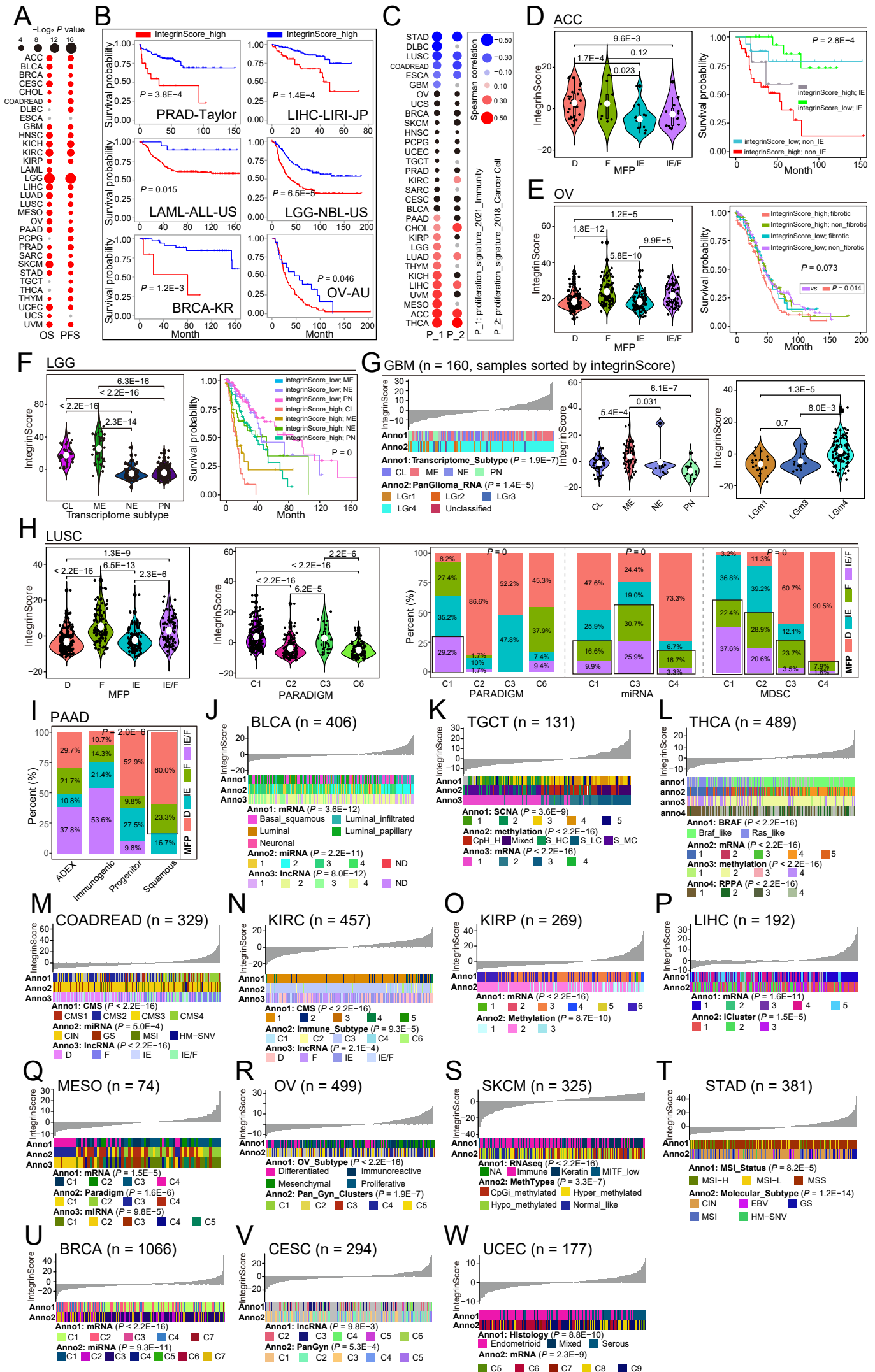

Supplement: qzae035_Supplementary_Data [file qzae035_supplementary_data.zip › Figure_S5 E.pdf]

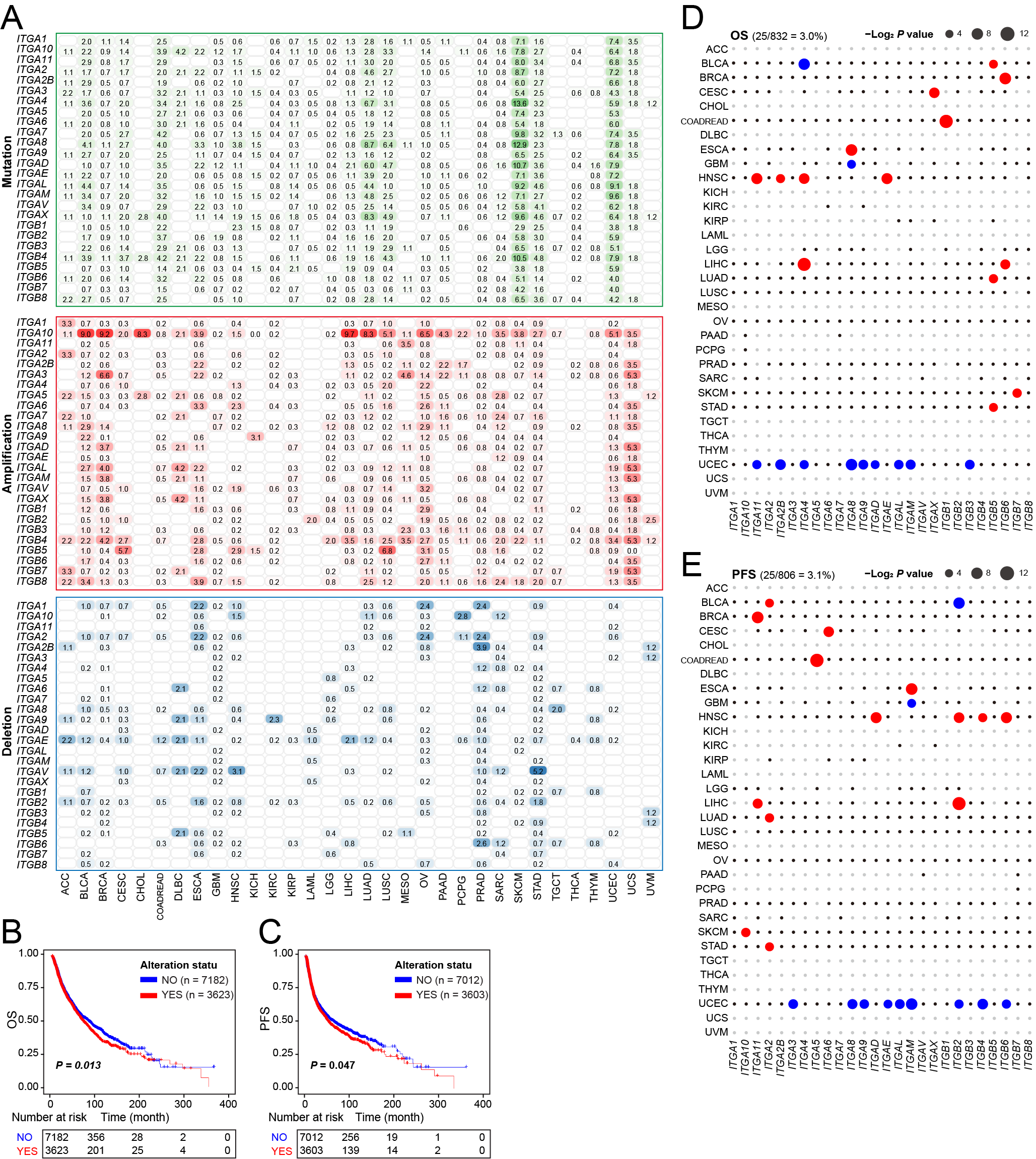

Supplement: qzae035_Supplementary_Data [file qzae035_supplementary_data.zip › Figure_S1.png]

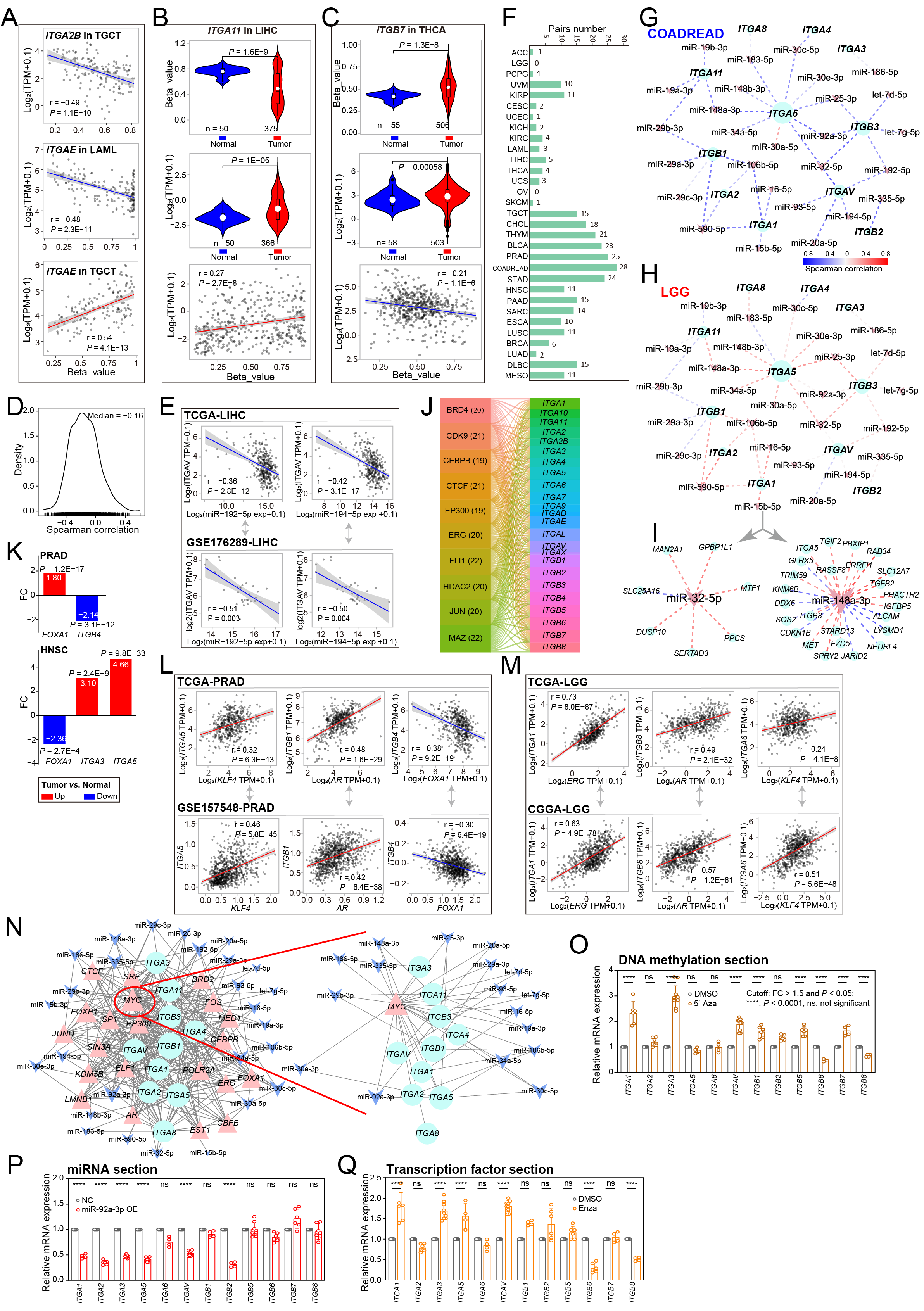

Supplement: qzae035_Supplementary_Data [file qzae035_supplementary_data.zip › Figure_S3.png]

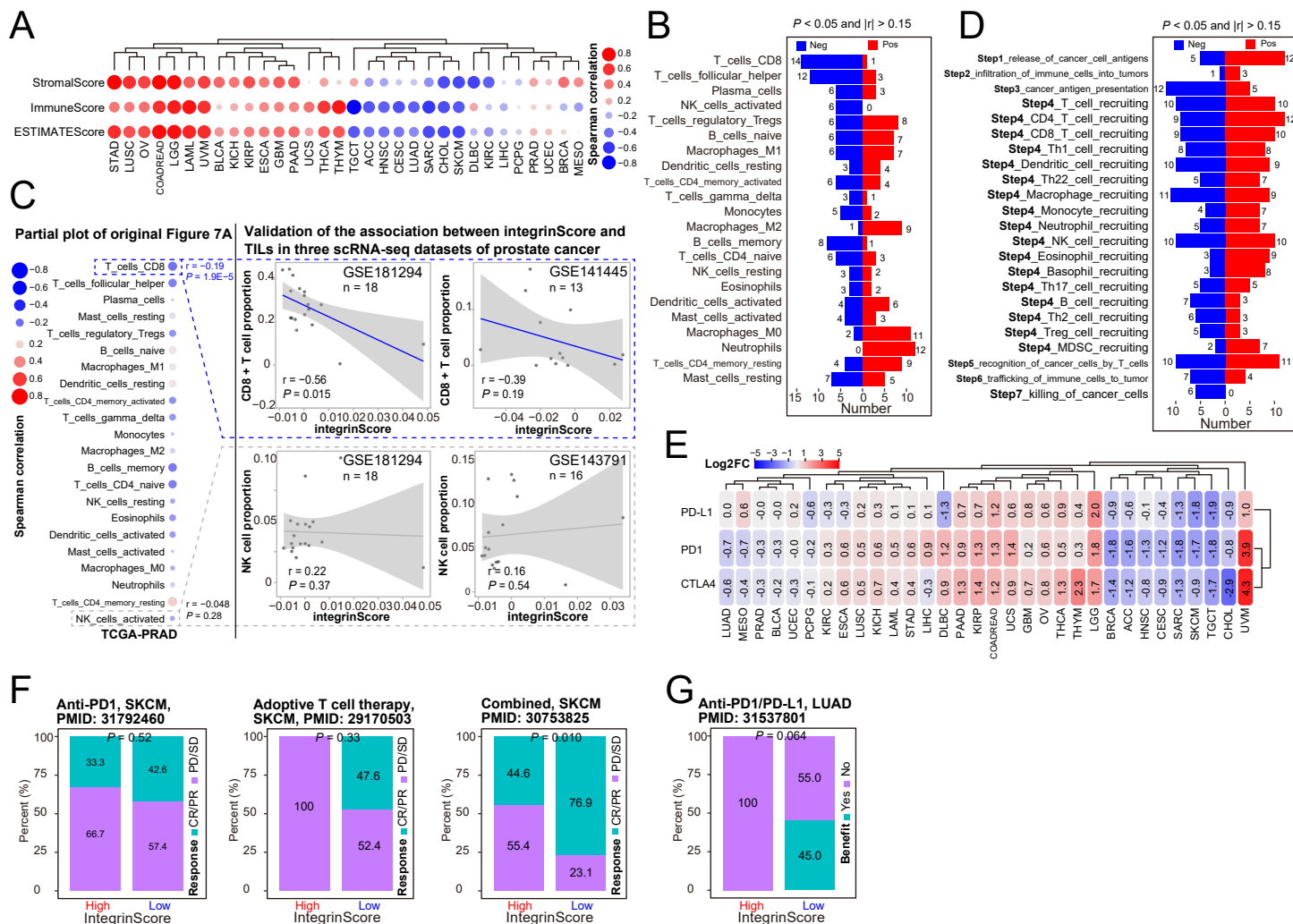

Supplement: qzae035_Supplementary_Data [file qzae035_supplementary_data.zip › Figure_S7 E.pdf]

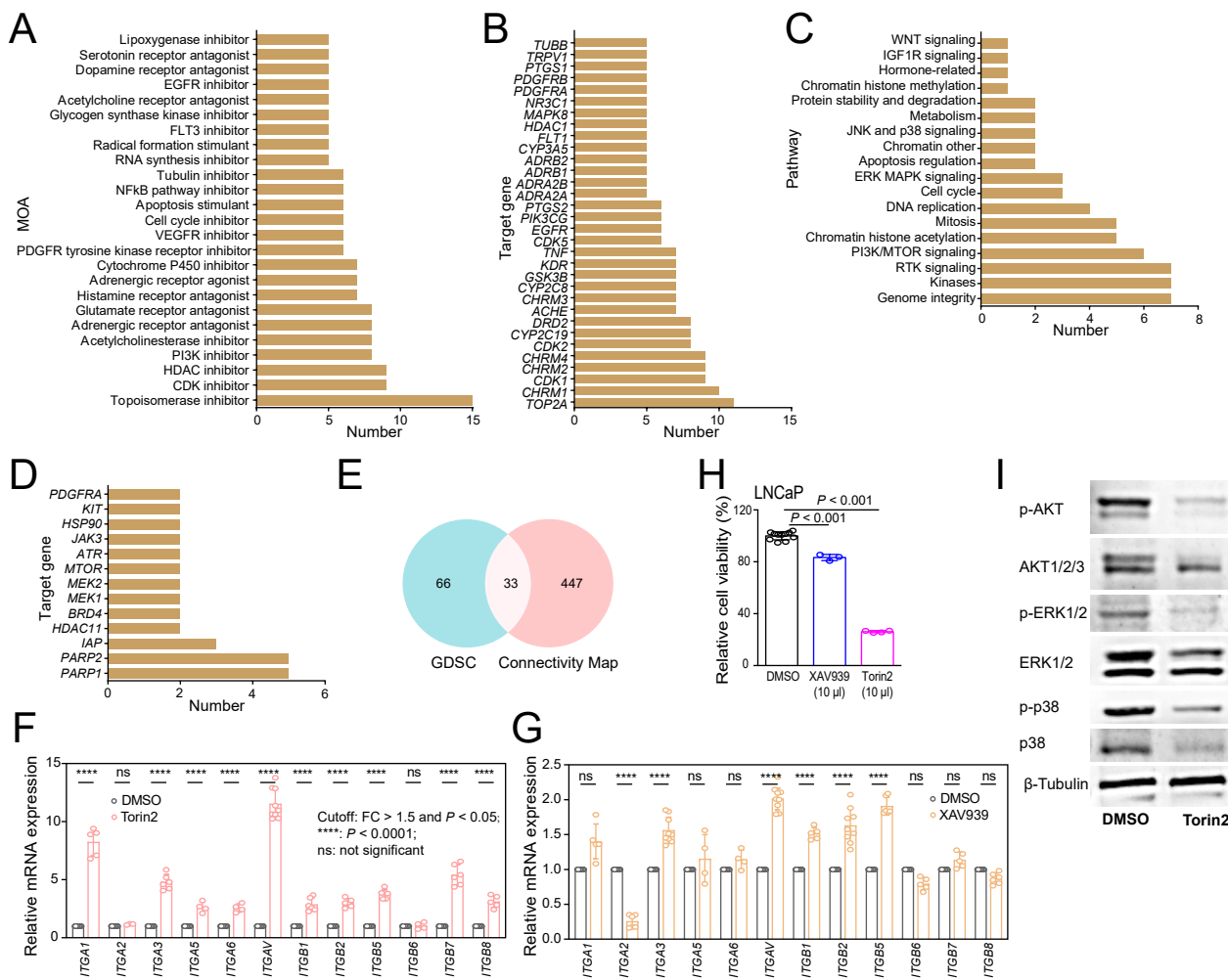

Supplement: qzae035_Supplementary_Data [file qzae035_supplementary_data.zip › Figure_S8 E.pdf]

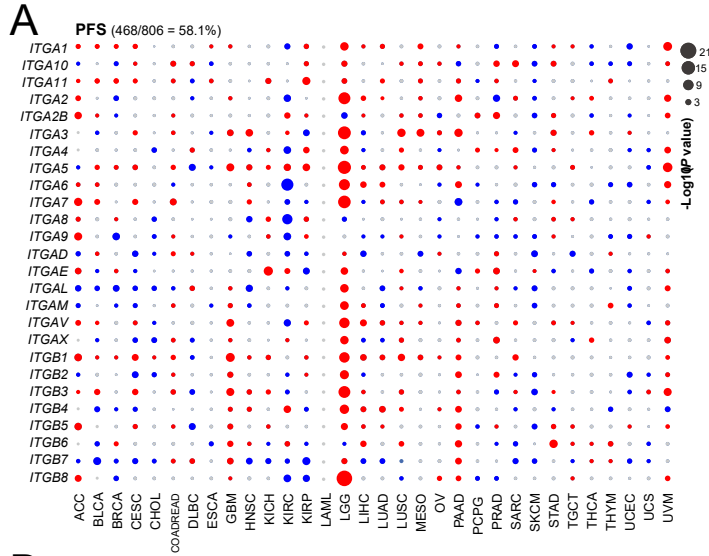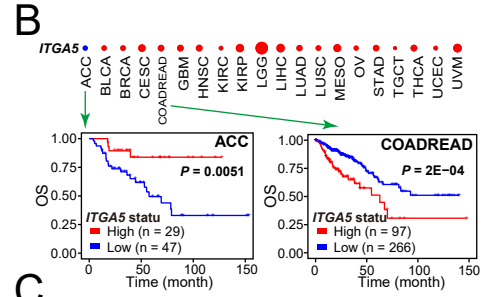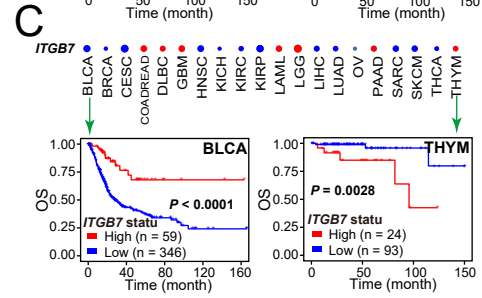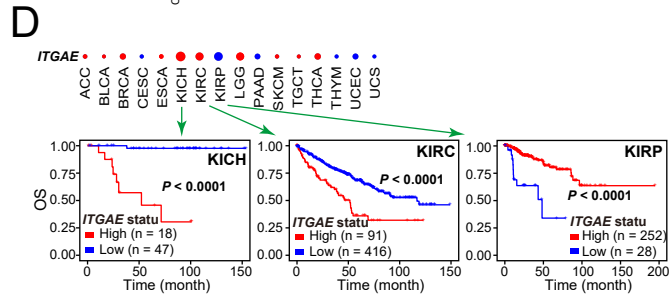

Supplement: qzae035_Supplementary_Data [file qzae035_supplementary_data.zip › Figure_S4 E.pdf]
